# Phylogenetic diversity, functional pathways, and network interactions of ocular chlamydia-like organisms (CLOs) in trachoma-endemic Ethiopia

**DOI:** 10.1128/mbio.00534-26

**Published:** 2026-04-23

**Authors:** Olusola Olagoke, Xinhe Zheng, Seongwon Chung, Hiwot Degineh Mengistie, Kaleb Asfaha, Timothy D. Read, Deborah Dean

**Affiliations:** 1 Division of Global Health and Infectious Diseases, Department of Pediatrics, University of California San Francisco School of Medicine 416977, San Francisco, California, USA; 2 Benioff Center for Microbiome Medicine, University of California San Francisco 8785, San Francisco, California, USA; 3 Bahir Dar Specialty Eye Center, Bahir Dar, Ethiopia; 4 School of Optometry & Vision Science, University of California Berkeley 115121, Berkeley, California, USA; 5 Division of Infectious Diseases, Department of Medicine, Emory University School of Medicine 12239, Atlanta, Georgia, USA; 6 Division of Global Health and Infectious Diseases, Department of Medicine, University of California San Francisco School of Medicine 166668, San Francisco, California, USA; 7 Department of Bioengineering, Joint Program, University of California San Francisco and University of California Berkeley 8785, San Francisco, California, USA; Universite de Geneve, Geneva, Switzerland

**Keywords:** Chlamydia-like organisms, trachoma, metagenomic shotgun sequencing, ocular microbiome, pathogenesis, Ethiopia

## Abstract

Trachoma is the leading infectious cause of blindness worldwide and classically attributed to C*hlamydia trachomatis* (Ct). However, other members of the phylum Chlamydiae, particularly environmental chlamydia-like organisms (CLOs), may modulate ocular ecology and influence disease outcomes. Here, we investigated CLO distribution, phylogeny, and microbiome associations among 1,059 individuals from trachoma-endemic communities in Ethiopia using targeted 16S rRNA sequencing and metagenomic shotgun sequencing. CLOs were detected in 249 (23.3%) participants of all ages and sexes and were significantly less likely to be associated with Ct or trachomatous scarring (TS) and trichiasis (TT). Phylogenetic analyses revealed extensive CLO diversity with six novel phylotypes, the most abundant of which was ancestral to *Sorochlamydiaceae*—a family linking pathogenic *Chlamydiaceae*, which includes the genus *Chlamydia*, and symbionts of protists. CLO-positive microbiomes exhibited significantly greater species richness and evenness with distinct differences in community composition relative to CLO-negative microbiomes. These effects were most pronounced among males and older adults. Functional profiling revealed widespread depletion of biosynthetic and metabolic pathways in CLO-positive microbiomes, particularly in participants with TS/TT, suggesting reduced community biosynthetic capacity and niche modification. Species interaction network analyses demonstrated substantial reorganization of microbial associations in the presence of CLOs with increased connectivity and centrality compared to CLO-negative networks. These findings identify CLOs as prevalent, phylogenetically diverse, and ecologically influential members of the microbiome. Their inverse association with Ct and TS/TT underscores the importance of considering intracellular symbionts beyond Ct in understanding conjunctival microbial ecology, resilience, and trachoma pathogenesis and for designing novel control strategies.

**IMPORTANCE:**

Trachoma caused by *Chlamydia trachomatis* (Ct) remains the leading infectious cause of blindness globally. While control efforts focus exclusively on Ct, other members of the phylum Chlamydiae, such as chlamydia-like organisms (CLOs), inhabit mucosal surfaces but remain understudied in the eye. Using targeted 16S rRNA and metagenomic shotgun sequencing of conjunctival samples from villagers in trachoma-endemic Ethiopia, CLOs were prevalent (23.3%; 249/1,059), phylogenetically diverse, including novel Chlamydiae phylotypes, and inversely associated with both Ct infection and severe scarring disease. CLO microbiomes had increased microbial diversity, altered community composition, depleted metabolic pathway abundance, and reorganized species interaction networks compared to CLO-negative microbiomes. These findings challenge the singular focus on Ct in trachoma control and research and suggest that CLOs represent ecologically significant members of the conjunctival microbiome. Further research on their interactions with ocular microbial communities could reveal new insights into trachoma pathogenesis and inform more holistic approaches to disease control.

## INTRODUCTION

Trachoma remains the leading infectious cause of blindness worldwide with the highest burden of disease concentrated in sub-Saharan Africa, particularly the Amhara region of Ethiopia (1). The pathogenesis of trachoma has traditionally been attributed to *Chlamydia trachomatis* (Ct), although evidence from Nepal and Guinea indicates that other species of the genus *Chlamydia*, such as *C. psittaci*, may be involved (2–4). Additional studies suggest that other members of the phylum Chlamydiae outside the known human and animal pathogens, colloquially referred to as “chlamydia-like organisms (CLOs).” (5) may also play an unrecognized role in ocular health and disease (6, 7). CLOs are a diverse group of obligate intracellular Chlamydiae that are widely detected in environmental reservoirs and free-living amoebae, commonly referred to as environmental Chlamydiae or chlamydia-related bacteria (8, 9). They are distributed across several families, including *Parachlamydiaceae*, *Rhabdochlamydiaceae*, *Simkaniaceae*, and *Waddliaceae*. CLOs have also been identified in human clinical specimens from respiratory, reproductive, and ocular sites (10–14). In trachoma-endemic settings, CLOs have been detected in conjunctival swabs from individuals with and without clinical signs of disease (10), raising questions about their potential contribution to ocular microbiome composition, immune modulation, and disease outcomes.

The ocular surface harbors a diversity of organisms that may influence susceptibility to Ct infection and disease severity (15, 16). Shifts in microbiome composition and diversity have been associated with trachoma, particularly in relation to Ct infection (17). However, the role of CLOs in shaping these microbial communities and their potential interactions with Ct remains poorly understood. Given that CLOs are phylogenetically related to Ct and share similar intracellular lifestyles, they may compete for host niches and resources, modulate immune responses, or alter microbiome community function or structures in ways that influence trachoma pathogenesis. Profiling microbial compositions at the species level offers a powerful framework for summarizing microbiome configurations, while microbial species co-occurrence networks provide further resolution on potential ecological interactions (18). Integrating these approaches may help determine whether CLO presence corresponds to distinct microbial community structures or altered species interaction patterns, offering mechanistic insights into their ecological and functional roles.

Here, we investigated the prevalence, diversity, predicted functional metabolic pathways, and network interactions of CLOs in the conjunctival microbiomes of a large, well-characterized trachoma-endemic population in Amhara, Ethiopia. Using molecular detection, phylogenetic analysis, and microbiome profiling, we examined the distribution of CLOs, their associations with demographic and clinical variables, and their potential influence on microbial diversity, community composition, function, and networks. Our study provides new insights into the ecology of CLOs in the ocular environment and their potential role in trachoma-endemic communities.

## RESULTS

### Chlamydiae-like organisms (CLOs) are significantly less likely to be associated with *C. trachomatis* infection or trachoma grade TS/TT

**Fig 1:**
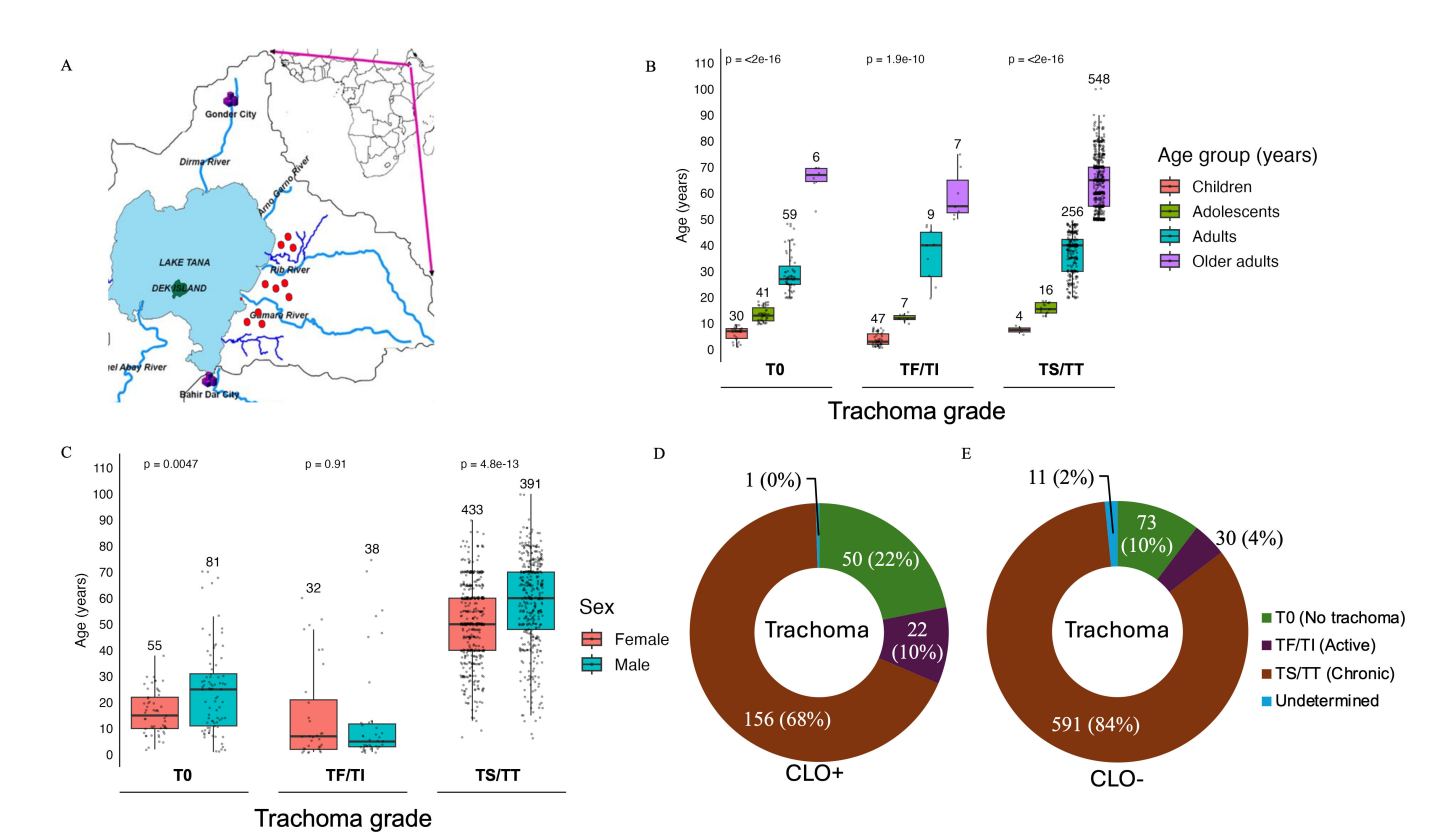
Study population characteristics. (**A**) Map of study regions in northwestern Ethiopia showing the locations of villages in Amhara that were sampled for this study. Red dots indicate the 10 villages from which conjunctival samples were collected. (**B**) Age distribution of study participants across trachoma grades. The number above each bar in the graph shows the total number of participants for each age group. Horizontal lines depict median age. Distribution of trachoma grades is shown across (**C**) sex and by (**D**) CLO-positive and (**E**) CLO-negative individuals.

The study population comprised 1,059 individuals enrolled after informed consent. Participants were from three regional clusters of three, four, and three villages, respectively (Fig. 1A), within the Amhara region. Table 1 shows the bivariate analysis of the association of CLOs with age group, gender, trachoma grade, and *Ct* infection. Clinical grading of trachoma was performed for all participants: no trachoma (T0), active trachoma (TF/TI), and chronic trachoma (TS/TT) (see Materials and Methods). There was no significant association of CLO with gender. Those with a CLO were significantly less likely to be infected with Ct (odds ratio [OR] = 0.55, 95% confidence interval [CI] = 0.33–0.91, *P* < 0.05) or have TS/TT (OR = 0.42, 95% CI = 0.29–0.62, *P* < 0.0001). For adolescents, the majority who had a CLO did not have acute or chronic trachoma and were not infected with Ct. The distribution of trachoma grade by age group is shown in Fig. 1B, by sex in Fig. 1C, and by CLO status in Fig. 1D and E.

**TABLE 1:** Bivariate analysis of the association of CLOs with age group, gender, trachoma grade, and *C. trachomatis* infection status^*a*^

Parameter	Total (*n* = 1,059)	No. (%)		OR	95% CI	*P* value
		CLO− (*n* = 810 [76.5%])	CLO+ (*n* = 249 [23.5%])			
Age (years; median 50 years)						
Children (<10)	82	59 (72.0)	23 (28.0)	0	Ref	
Adolescents (10–19)	64	36 (56.3)	28 (43.8)	**2.01**	**1.00–4.03**	**0.048**
Adults (20–49)	329	248 (75.8)	81 (24.2)	0.82	0.46–1.45	0.522
Older adults (>49)	577	460 (79.7)	117 (20.3)	0.64	0.37–1.11	0.109
Missing	7	7 (100)				
Sex						
Female	531	408 (76.8)	123 (23.2)	0	Ref	
Male	526	400 (76.0)	126 (24.0)	1.04	0.78–1.39	0.762
Missing	2	2 (100)				
Trachoma grade						
T0	137	86 (62.8)	51 (37.2)	0	Ref	
TF/TI	70	44 (62.9)	26 (37.1)	1	0.55–1.81	1
TS/TT	840	669 (79.6)	171 (20.4)	**0.42**	**0.29–0.62**	**<0.0001**
Missing	12	11 (91.7)	1 (8.3)			
*C. trachomatis* infection						
Negative	933	704 (75.5)	229 (24.5)	0	Ref	
Positive	126	107 (84.9)	19 (15.1)	**0.55**	**0.33–0.91**	**0.019**

^*a*^ Odds ratios are relative to the first category in each subgroup. Bold text represents statistically significant comparisons.

### Some CLOs are phylogenetically similar to four different Chlamydiae families, but many are unclassified, and the most abundant phylotype is ancestral to *Sorochlamydiaceae*

**Fig 2:**
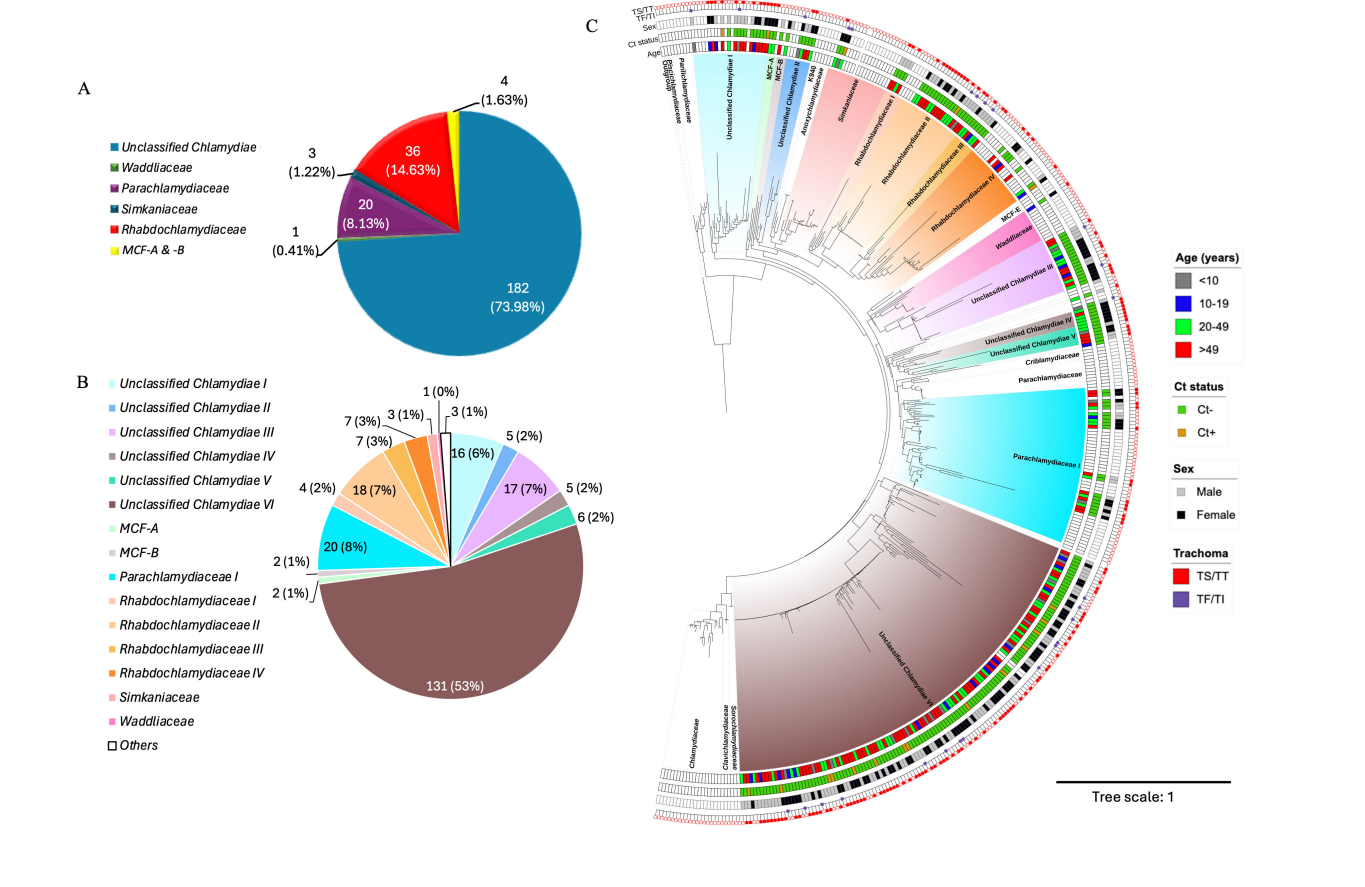
Diversity of CLOs based on 16S rRNA sequences in Amhara, Ethiopia at the (**A**) known Chlamydiae family and (**B**) phylotype levels. (**C**) Phylogenetic relationship between the CLOs identified in this study with previously published members of the Chlamydiae phylum. Non-colored boxes for the four categories of age, Ct status, sex, and trachoma grade represent publicly available sequences. Colored clusters show phylotypes with representative members from the current study. Identical color scales are used in panels **B and C**.

The 16S rRNA sequence of each CLO sample was aligned against 157 publicly available CLO sequences in National Center for Biotechnology Information (NCBI) (using BLASTn) (19), ENA (20), and ChlamDb (21). Only 60 (24.1%) of the 249 CLO sequences in this study fell within known CLO families: *Parachlamydiaceae* (*n* = 20; 8.13%), *Rhabdochlamydiaceae* (*n* = 36; 14.63%), *Simkaniaceae* (*n* = 3; 1.22%), and *Waddliaceae* (*n* = 1; 0.41%). Most samples were unclassified Chlamydiae (*n* = 182; 73.98%) with four other samples classified as MCF-A and -B (1.63%) (Fig. 2A).

To further resolve the classification of our sequences, we used TreeCluster, a phylogenetic relationship tool (22), to identify phylotype clusters on phylogenetic trees (see Materials and Methods). Using available CLO sequences (*n* = 157) from the databases above, which represent all known Chlamydiae family members, the unclassified Chlamydiae sequences were placed into six phylotypes: Unclassified Chlamydiae I–VI (Fig. 2B and C). The Unclassified Chlamydiae VI phylotype, which accounts for 53% (*n* = 131) of all CLOs in this study, was ancestral to *Sorochlamydiaceae* (23–25), from which the *Chlamydiaceae* family evolved that includes the genus *Chlamydia* (Fig. 2C). Four distinct phylotypes were identified within *Rhabdochlamydiaceae* (Fig. 2C). Of the 19 participants with both CLO and Ct infection, 14 were in Unclassified *Chlamydiaceae* VI, with the remaining five in Unclassified Chlamydiae I (*n* = 2), *Rhabdochlamydiaceae* II (*n* = 1) and IV (*n* = 1), and *Simkaniaceae* (*n* = 1) phylotypes (Fig. 2C).

We also observed what appears to be clonal expansions within several phylotypes with monophyletic clades, including *Rhabdochlamydiaceae* II (nested within the *Rhabdochlamydiaceae* family), Unclassified *Chlamydiaceae* III (more closely related to the *Waddliaceae* family), and Unclassified Chlamydiae VI (Fig. 2C). These clades exhibited dense clusters of closely related sequences with short internal branch lengths, consistent with recent diversification from a common ancestor, although sampling may have contributed to this.

There was no significant association between CLO phylotype and age group, gender, or Ct status. However, both bivariate and multivariate analyses showed that participants with Unclassified Chlamydiae III and Unclassified Chlamydiae VI were significantly less likely to present with TS/TT (Table S1). In multivariate analysis adjusting for age, sex, and Ct status, the inverse association with TS/TT remained significant for both Unclassified Chlamydiae III (adjusted OR = 0.25, 95% CI: 0.09–0.71, *P* = 0.009) and Unclassified Chlamydiae VI (adjusted OR = 0.46, 95% CI: 0.29–0.74, *P* = 0.001).

### Ocular microbiomes with CLOs have a greater evenness and richness of microbes compared to microbiomes without CLOs

The same DNA samples prepared for metagenomic shotgun sequencing (MSS) had also been used for targeted 16S rRNA amplicon sequencing. Of the 249 samples from individuals with CLOs, 212 had sufficient DNA yield to perform MSS. After depletion of host and contaminant reads using stringent contaminant filtering, given the low microbial biomass of conjunctival samples (see Materials and Methods), 98 (46.2%) of 212 had at least one read that matched to a CLO (range: 1–30; mean: ≈4.3). This is not surprising because CLOs represent a much lower component of the microbiome than many bacteria, including Ct.

**Fig 3:**
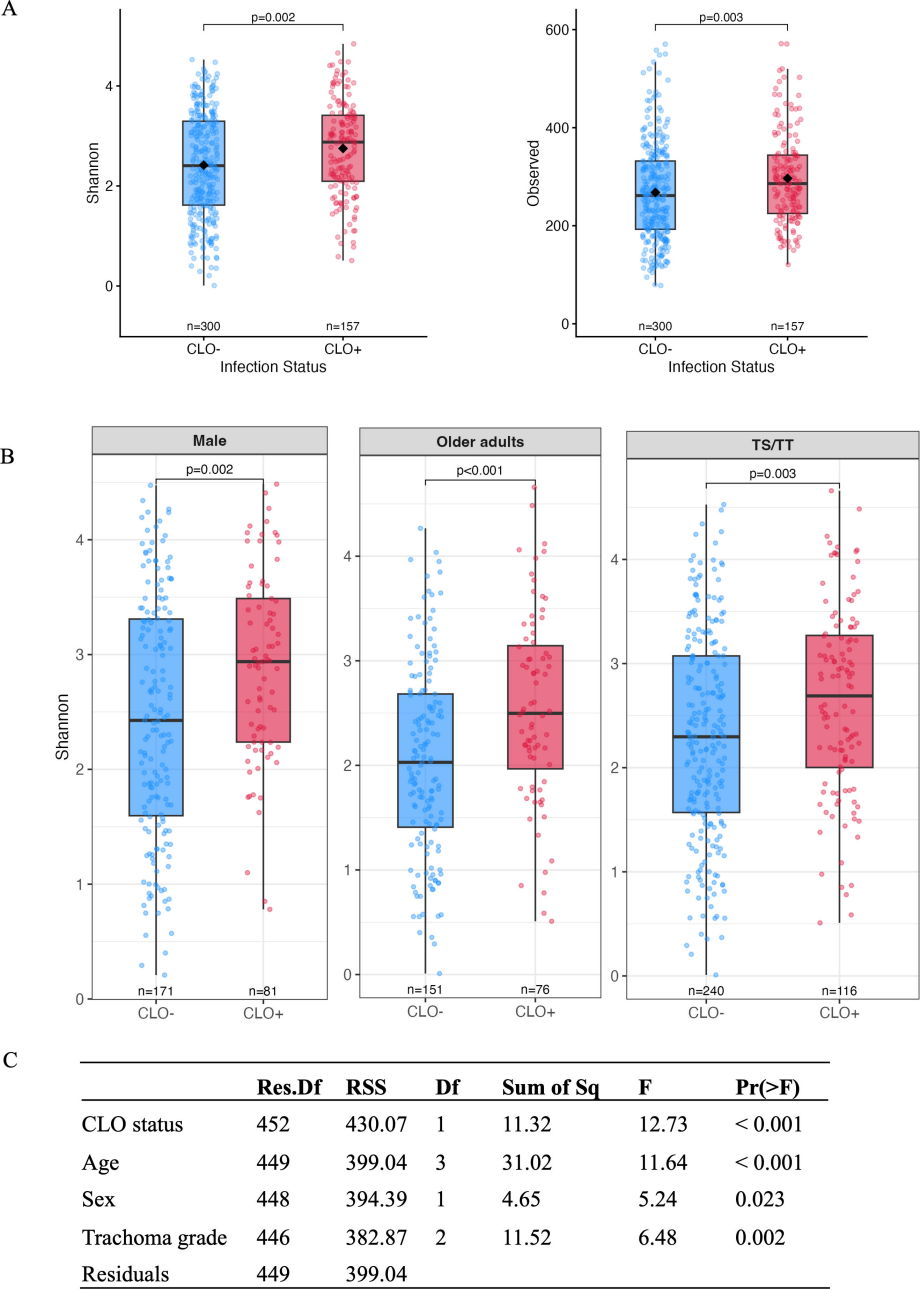
Alpha diversity metrics showing (**A**) comparison of observed species and Shannon index among CLO-negative and -positive individuals. (**B**) Stratified comparisons of Shannon diversity by sex (males), age group (older adults), and trachoma grade (TS/TT). Boxplots represent the median and interquartile range; points denote individual samples. Only statistically significant comparisons are shown. Complete stratified results for both observed species richness and Shannon diversity are shown in Fig. S1. (**C**) Multivariable ANOVA results demonstrating that CLO status is independently associated with Shannon diversity after adjustment for age group, sex, and trachoma grade. Abbreviations: Res.Df, residual degrees of freedom; RSS, residual sum of squares; Df, degrees of freedom (number of parameters for each variable); Sum of Sq, sum of squares attributable to each variable; and F, F-statistic; Pr(>F), *P* value. Residuals represent the unexplained variance remaining after accounting for all model predictors.

To understand the role of CLOs in the microbiome without the confounding effects of Ct, only Ct-negative microbiomes were included in microbiome analyses. Overall, CLO-positive individuals exhibited a significantly higher number of observed species compared with CLO-negative individuals (Fig. 3A). Analyses stratified by age, sex, and trachoma grade showed consistently higher observed species richness in CLO-positive males and females as well as in individuals with TS/TT (Fig. S1). Importantly, the Shannon index was significantly higher in CLO-positive compared to CLO-negative individuals (Fig. 3A). Stratified analyses further showed significantly higher Shannon indices in CLO-positive older adults, males, and individuals with TS/TT compared to CLO-negative individuals (Fig. 3B). In a multivariable analysis of variance (ANOVA) model, CLO status was independently associated with Shannon diversity after adjustment for age group, sex, and trachoma grade, with CLO status exhibiting the strongest standardized effect [*F*(1, 452) = 12.73, *P* < 0.001], supporting a major role for CLOs in shaping microbiome diversity (Fig. 3C).

Beta diversity was used to evaluate whether overall microbial community composition differed between CLO-positive and -negative microbiomes. Using permutational multivariate analysis of variance (PERMANOVA) on Bray-Curtis dissimilarities, significant segregation of microbiomes was observed across multiple demographic and clinical categories, including in the overall cohort (*P* = 0.001) comparing CLO-positive to -negative participants as well as in multiple subgroups defined by age, sex, and trachoma grade. These included adult males, older adult males with TS/TT, and older adult females (Fig. S2).

### CLO-positive microbiomes are associated with differences in microbial community composition and species abundance distinct from CLO-negative microbiomes

**Fig 4:**
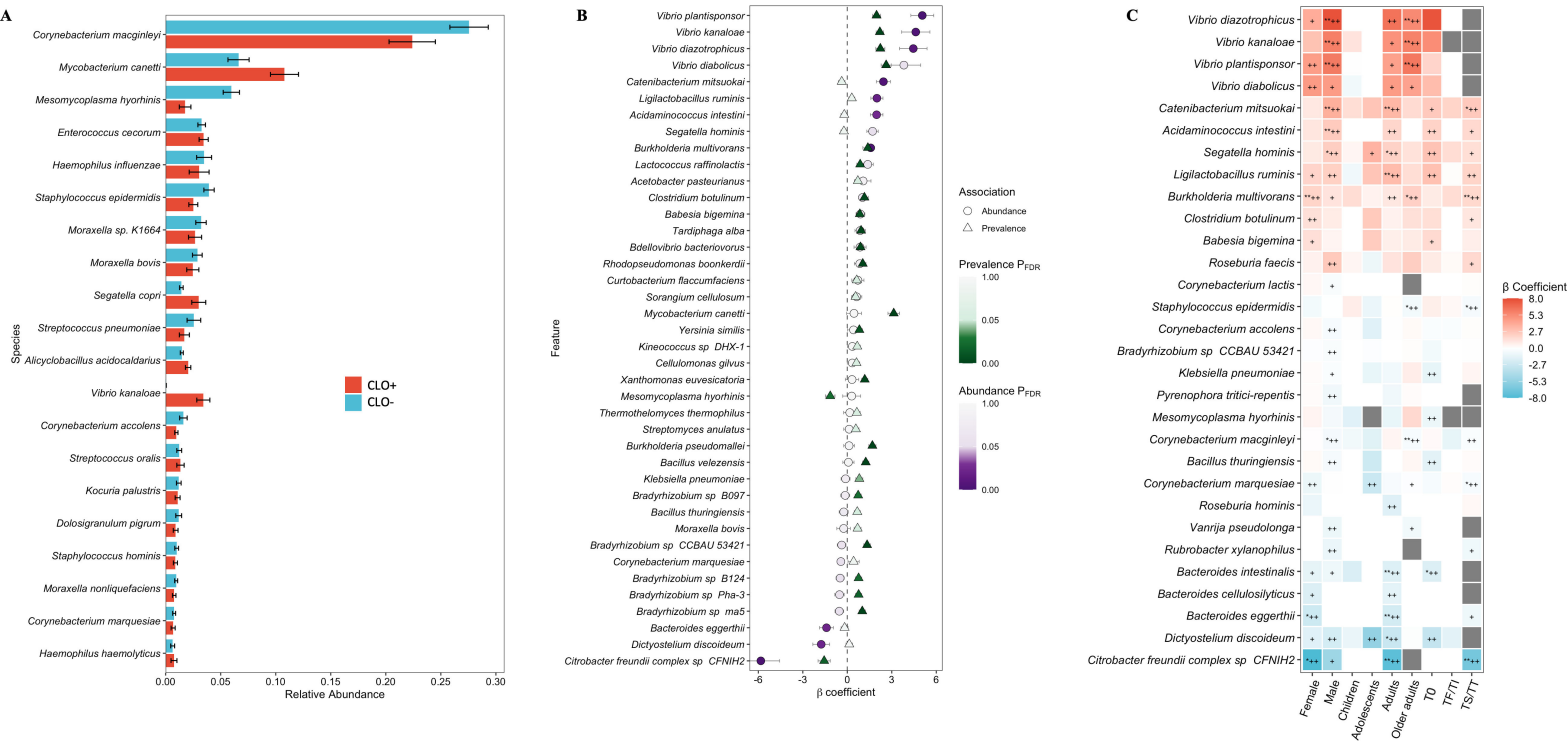
Relative abundance and differentially abundant species in CLO-associated microbiomes. (**A**) Overall relative abundance of the top 20 microbial species in CLO-positive and -negative microbiomes without controlling for covariates. Bars show mean relative abundance, with error bars representing the standard error. (**B**) Forest plot of species significantly associated with CLO status (MaAsLin3, *q* < 0.1), showing abundance (circles) and prevalence (triangles) associations. β coefficients represent log2 fold change (CLO-positive vs CLO-negative). (**C**) Stratified analysis showing CLO effect within tested groups defined by sex, age group, and trachoma grade while adjusting for remaining covariates. Red, enriched in CLO-positive microbiomes; blue, depleted in CLO-positive microbiomes. Asterisks denote FDR-adjusted significance (*q*-value): *, *q* < 0.1; **, *q* < 0.05; +, *P* < 0.05; ++, *P* < 0.01. Gray = NA.

The relative abundance profiles of microbial species differed between CLO-positive and -negative microbiomes, indicating systematic differences in community structure associated with CLO status (Fig. 4A). As above, only Ct-negative microbiomes were included in these analyses to avoid any confounding effects from Ct. To quantify differences while accounting for other potential confounding factors like age group, sex, and trachoma grade, we employed MaAsLin3 (26), which fits separate models for abundance (how much) and prevalence (presence/absence). This approach enabled simultaneous modeling of multiple covariates and robust identification of taxa independently associated with CLO status. Multiple taxa (*n* = 19), including *Vibrio plantisponsor*, *V. kanaloa*, *V. diabolicus*, *Burkholderia multivorans*, and *Lactococcus raffinolactis*, were significantly associated with CLOs where positive *β* coefficients indicated increased abundance or prevalence in these microbiomes (Fig. 4B). Conversely, several taxa (*n* = 9), including *Citrobacter freundii*, *Dictyostelium discoideum*, and *Bacteroides eggerthii*, showed negative associations with reduced abundance in CLO-positive microbiomes (Fig. 4B). A complete list of statistically significant species is shown in Table S2.

To assess the stability of these associations across host subgroups, we performed stratified MaAsLin3 (26) analyses, testing CLO-positive versus -negative microbiomes separately within strata defined by sex, age group, and trachoma grade while adjusting for remaining covariates. Several CLO-associated taxa retained consistent directions of association across strata. Although effect sizes varied, the overall patterns were concordant with the full-cohort model, indicating that the same CLO-related microbial signatures were largely preserved across demographic and clinical subgroups (Fig. 4C).

### CLO-associated ocular microbiomes exhibit predicted functional depletion of core metabolic pathways

**Fig 5:**
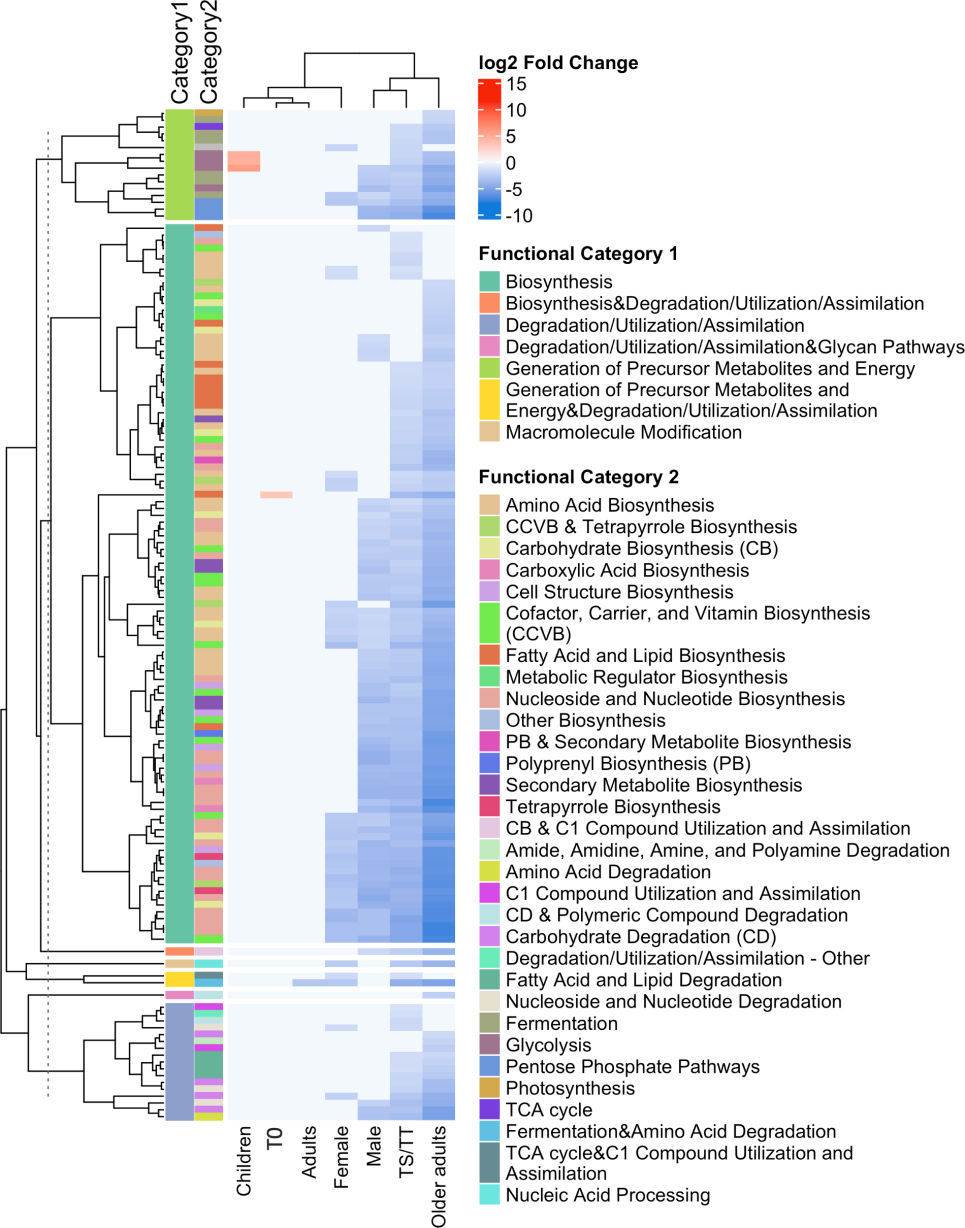
Differential abundance of functional pathways comparing CLO-positive and -negative microbiomes across subgroups, annotated by two hierarchical functional categories. The heatmap shows the log_2_ fold change in pathway abundance between CLO-positive and -negative associated microbiomes across clinical subgroups stratified by age group, sex, and trachoma grade, with adjustment for remaining covariates within each stratum. Pathways (*y*-axis) are clustered hierarchically and grouped by Functional Category 1 (broad metabolic class), with color-coded side bars indicating both functional categories 1 and 2 (specific functional subclass within the broad metabolic classes). Clinical subgroups are shown on the *x*-axis, with hierarchical clustering based on similarity in pathway abundance profiles. Blue to red color scale represents log_2_ fold changes, with blue indicating depletion and red indicating elevation in CLO-positive microbiomes. Legends to the right indicate color assignments for each functional category. A detailed description of all indicated pathways for each subgroup is available in Fig. S3.

To investigate whether CLOs are associated with distinct functional metabolic pathway profiles in the ocular microbiome, we analyzed community-level pathway abundance derived from HUMAnN (27), again controlling for Ct infection. Relative abundance was log-transformed and modeled using linear regression stratified by clinically relevant subgroups (i.e., sex, age group, trachoma grade, and their interactions). This enabled identification of statistically significant CLO-associated functional pathways inferred from taxonomic composition rather than measured metabolites compared to CLO-negative pathways while controlling for demographic and clinical trachoma grade variability across the population. Across all clinical subgroups, 98.9% of the significant pathway changes reflected decreases in CLO-positive microbiomes. These included pathways involved in nucleotide metabolism (e.g., inosine-5′-phosphate biosynthesis, UMP biosynthesis, and pyrimidine salvage), amino acid biosynthesis (e.g., L-lysine, L-methionine, and L-arginine), cofactor and vitamin biosynthesis (e.g., tetrapyrrole biosynthesis and phosphopantothenate biosynthesis), and central carbon metabolism (e.g., pentose phosphate pathway and methylerythritol phosphate pathway) (Fig. 5; Fig. S3). Only four pathways were significantly increased in CLO-positive microbiomes: glycolysis IV; glycolysis I (from glucose 6-phosphate); and glycolysis II (from fructose 6-phosphate) in children overall; and gondoate biosynthesis (anaerobic) among those with T0 overall.

Trachoma-related subgroup comparisons with statistically significant pathway changes were from participants with TS/TT, and in every case, multiple pathways were decreased in CLO-positive relative to CLO-negative microbiomes (Fig. 5; Fig. S3). The depleted pathways span a broad range of functional categories, notably amino acid biosynthesis (e.g., L-lysine and L-methionine), carbohydrate degradation (sucrose, glucose, fructose pathways), nucleotide metabolism (e.g., purine and pyrimidine biosynthesis), energy metabolism (glycolysis, fermentation), and cofactor biosynthesis (folate, heme, molybdopterin) (Fig. 5; Fig. S3). Contributing microbial species driving these predicted functional differences between CLO-positive and -negative microbiomes across subgroups for each pathway are shown in Table S3. For example, the observed increase in glycolysis (from glucose 6-phosphate) in CLO-positive children was largely driven by contributions from *Strepto*c*occus pneumoniae*, *Strepto*c*occus sinensis*, *Pantoea vagans*, and *P. agglomerans* (Table S3A).

### CLO-positive and -negative microbiomes have distinct microbial co-occurrence networks

**Fig 6:**
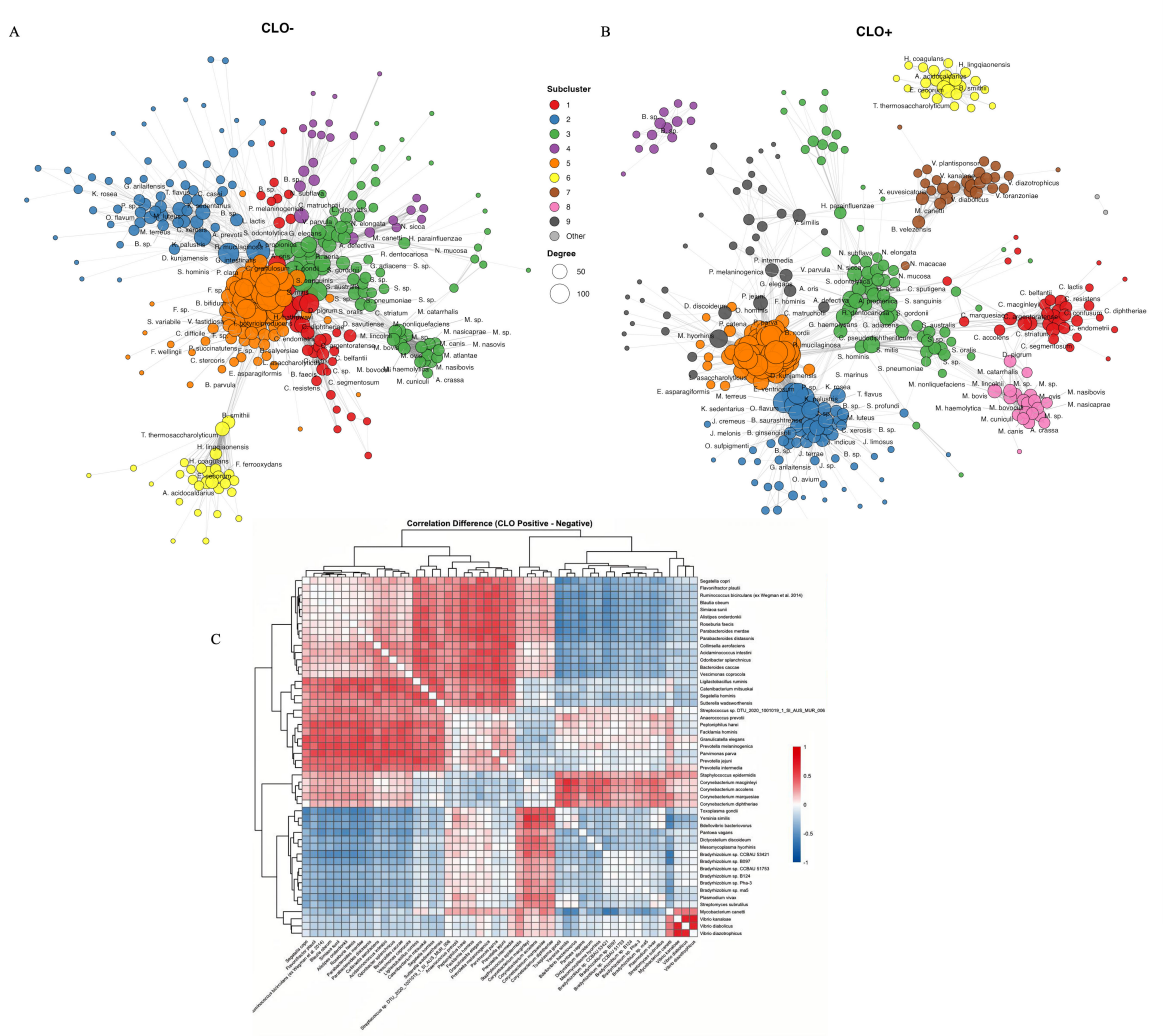
Co-occurrence networks of dominant microbial species by CLO status. SparCC correlation networks for (**A**) CLO-negative and (**B**) CLO-positive microbiomes. Nodes represent species with node size proportional to degree (number of connections). Node colors indicate subclusters identified by Louvain community detection. Gray nodes indicate species in subclusters with fewer than three members ("other"). Edges (lines connecting nodes) represent significant correlations (|*r*| > 0.3). Species labels are shown for top hub species (highly connected species) per subcluster. (**C**) Differential correlation heatmap showing the difference in SparCC correlations between CLO-positive and -negative networks for the top 50 species ranked by total absolute correlation difference. Red indicates species pairs with stronger positive correlations in CLO-positive microbiomes; blue indicates weaker or reversed correlations. Hierarchical clustering (Euclidean distance, complete linkage) groups species with similar differential correlation patterns. A complete list of all the species indicated in the co-occurrence networks is available in Table S4.

To further investigate the impact of CLOs on microbial community interactions, again controlling for Ct infection to avoid any confounding influence from Ct, we performed co-occurrence network analysis comparing CLO-positive and -negative microbiomes using SparCC (28). Networks were constructed using a correlation threshold of |*r*| > 0.3, and species were assigned to subclusters based on Louvain community detection (29). Despite differences in CLO status, both networks shared six core subclusters (Fig. 6A and B; Table S4). Subcluster 1 comprised canonical ocular surface commensals dominated by *Corynebacterium* species (i.e., *C. marquesiae*, *C. macginleyi*, *C. accolens*, and *C. striatum*) alongside *Staphylococcus epidermidis* and *Dolosigranulum pigrum*. Subcluster 2 contained skin-associated microbiota, including *Staphylococcus hominis*, *Micrococcus species*, *Kocuria species*, and *Dietzia kunjamensis*. Subcluster 3 contained oral and upper respiratory tract-associated taxa, including *Rothia mucilaginosa*, *Streptococcus* species (i.e., *S. mitis*, *S. oralis*, *S. sanguinis*, and *S. gordonii*), *Gemella haemolysans*, and *Neisseria* species. Subcluster 4 comprised soil-associated *Bradyrhizobium* species and *Mycobacterium canetti*. Subcluster 5 was the largest module in both networks, comprising predominantly gut-associated anaerobic bacteria, including *Roseburia* species (i.e., *R. intestinalis*, *R. faecis*, and *R. hominis*), *Bacteroides* species (i.e., *B. cellulosilyticus*, *B. eggerthii*, and *B. intestinalis*), *Ruminococcus bicirculans*, and *Alistipes onderdonkii* as hub species. Subcluster 6 occupied a peripheral network in both groups, containing thermophilic and environmental taxa, including *Thermoanaerobacterium thermosaccharolyticum* and *Alicyclobacillus acidocaldarius*. Table S4 contains the list of species in each subcluster.

Despite these conserved subclusters, CLO-positive and -negative networks exhibited marked structural differences. Three subclusters (i.e., 7–9) were exclusive to CLO-positive microbiomes (Fig. 6B). Most notably, subcluster 7 (27 species) was dominated by *Vibrio* species (i.e., *V. kanaloae*, *V. diabolicus*, and *V. plantisponsor*)—taxa significantly enriched in CLO-positive individuals by differential abundance analysis (Fig. 4C). Subcluster 8 (20 species) comprising *Moraxella* spp. (i.e., *M. lincolnii*, *M. nonliquefaciens*, and *M. catarrhalis*) was a distinct module, while subcluster 9 (39 species) contained *Mesomycoplasma hyorhinis* and oral anaerobes, including *Prevotella* species (i.e., *P. jejuni*, *P. intermedia*, and *P. melaninogenica*).

A differential SparCC correlation heatmap revealed clustered species with concordant co-occurrence changes between CLO-positive and -negative microbiomes (Fig. 6C). The most striking feature was the positive correlation block among *Vibrio* species (i.e., *V. kanaloae* and *V. diabolicus*) and *Mycobacterium canetti*, indicating strengthened co-occurrence in CLO-positive microbiomes corroborating emergence of subcluster 7 in the network analysis. Canonical ocular commensals (i.e., *Corynebacterium* species and *Staphylococcus epidermidis*) showed stable or slightly reduced correlations—consistent with the disrupted connectivity observed in subcluster 1.

## DISCUSSION

Our study demonstrates a substantial prevalence of CLOs in the trachoma-endemic population of Amhara, Ethiopia, with nearly a quarter (23.3%) of participants testing positive. The detection of CLOs across all four age groups, ranging from 20.3% in older adults to 43.8% in adolescents, and absence of a significant association with age or gender suggest a widespread distribution of these organisms within the community. Importantly, novel findings are the lack of a significant association between CLOs and Ct co-infection and TS/TT. This inverse association may have several explanations. One possibility is ecological competition or interference between CLO and Ct at the ocular surface, potentially leading to reduced CLOs in the presence of Ct infection or persistence. This may occur from an immune response that promotes inflammation and thereby eliminates or prevents CLO acquisition, similar to the inflammatory response of *Staphylococcus aureus* that removes all or most competitors during atopic dermatitis (30). It is also possible that Ct may outcompete CLOs through shared resource competition. Alternatively, it is possible that CLOs confer some level of cross-protection or immune effect against Ct infection and/or its sequelae, although we did not measure immune responses. Other host/environmental factors may also influence these interactions. Given that TS/TT usually develops in adulthood after repeated Ct infection in childhood (31), these findings may indicate that CLOs could alter the natural history of trachoma either through direct microbial network interactions (discussed below), competition, or possibly immunomodulation. Longitudinal studies incorporating immune and metabolite measurements along with network analyses are needed to tease out these temporal relationships.

Phylogenetic analysis revealed substantial diversity among CLOs detected in this population, spanning well-known families, such as *Parachlamydiaceae*, *Rhabdochlamydiaceae*, *Simkaniaceae*, and *Waddliaceae*, as well as a large proportion of novel phylotypes that are unclassified in the phylum Chlamydiae. This is consistent with previous environmental and clinical surveys where CLOs have been detected across diverse hosts and environments using 16S rRNA gene sequencing (6, 32). The predominance of unclassified Chlamydiae (nearly 74%) highlights the vast, understudied diversity within the phylum, echoing observations from recent metagenomic studies that indicate that the majority of Chlamydiae organisms remain undescribed (7, 8).

The application of the phylogenetic clustering tool, TreeCluster (22), enabled finer resolution of CLO diversity, revealing six distinct phylotypes of unclassified Chlamydiae. Unclassified Chlamydiae VI was the most abundant of the CLOs and ancestral to *Sorochlamydiaceae*, a recently discovered family that is an ancestral link between *Chlamydiaceae*—the family in which the genus *Chlamydia* resides—and symbionts of protists (23–25). Furthermore, 14 (73.7%) of the 19 Ct co-infections were in this phylotype, suggesting perhaps some permissive co-habitation, although there was no statistical association between Chlamydiae VI and Ct infection. In addition, Chlamydiae VI, along with Chlamydiae III, appeared to drive the reduced likelihood of having TS/TT, indicating that not all phylotypes confer the same effects. Our findings may, therefore, start to explain the complex evolutionary relationships within Chlamydiae and the novel lineages that may play distinct ecological roles in the ocular environment (33). Longitudinal studies are needed to further explore these relationships.

Microbiome profiling revealed that CLO-positive individuals exhibited significantly greater microbial alpha diversity compared to their CLO-negative counterparts. This pattern persisted across several subgroups, including males, older adults, and individuals with TS/TT, and remained robust after adjusting for demographic and clinical covariates in regression models. The concurrent increase observed in both richness and evenness indicates that CLO-positive conjunctivae harbor not only more species but also a more even distribution of taxa. CLO presence is, therefore, associated with a more complex microbial ecosystem, potentially reflecting altered ocular surface conditions that permit colonization by a broader range of microorganisms. Differential abundance analyses revealed consistent CLO-associated microbial signatures, with certain taxa recurrently enriched or depleted in CLO-positive individuals, irrespective of demographic or clinical trachoma status. Notably, various *Vibrio* species, *Catenibacterium mitsuokai*, *Ligilactobacillus ruminis*, *Acidaminococcus intestini*, *Burkholderia multivorans*, and *Clostridium botulinum* were enriched in CLO-positive microbiomes, while taxa, such as *Citrobacter freundii*, *Dictyostelium discoideum*, and *Bacteroides eggerthii*, were depleted. These findings suggest that CLOs may be linked to distinct community profiles that may also support the presence of CLOs. The observed depletion of known ocular and systemic pathogens, such as *Citrobacter freundii* (34, 35), also raises questions about the broader ecological and clinical implications of CLOs. It is possible that CLOs may compete with, or even inhibit, the infection or persistence of these pathogens (17). Conversely, the enrichment of certain taxa, such as *C. mitsuokai*, *L. ruminis*, *A. intestini*, and *Vibrio* spp., warrants further investigation to assess co-infection and pathogenic risk (36–38), especially as these organisms can arise under certain adverse conditions like malnutrition, poor hygiene, and immune suppression, and where infectious disease burdens are already high among trachoma-endemic communities (36, 39, 40).

Predicted functional pathway analysis showed that CLOs are associated with differences in the metabolic potential of the ocular microbiome. Across all clinical subgroups, the majority of differentially abundant pathways were depleted in CLO-positive compared to CLO-negative microbiomes. These pathways included nucleotide metabolism, amino acid biosynthesis, central carbon metabolism, and cofactor/vitamin production, although metabolic output may differ from these metagenome-based predictions. Such widespread depletion suggests that CLOs may be linked to an overall reduction in community functional capacity, potentially reflecting niche modification, resource limitation, or competitive exclusion (41, 42). Notably, the most consistent and pronounced functional depletion occurred in CLO-positive participants with TS/TT. This could reflect a community-wide shift toward a less metabolically versatile state, possibly as a consequence of host immune activation (43) and/or the direct effects of CLOs and co-existing pathogens. Similarly, reduced functional capacity has been observed in patients with Crohn’s and inflammatory bowel disease that contributes to impaired resilience and altered host-microbe interactions (44–46).

The species-level pathway contribution profiles further suggest that depleted taxa in CLO-positive microbiomes may play key roles in shaping the community’s metabolic landscape. For example, reduced pathway contributions from metabolic generalists, such as *Klebsiella pneumoniae* and *Staphylococcus epidermidis*—both of which possess complete gluconeogenic capacity—could underlie the observed depletion of gluconeogenesis-related functions in participants with TS/TT (47–49). Given the obligate intracellular lifestyle and metabolic auxotrophy of Chlamydiae and related taxa (50), it is plausible that their presence within the community may drive a reduced overall biosynthetic potential, but allow evenness in the overall species relative abundance. Future metatranscriptomic or metabolomic studies are needed to validate these predictions.

We also found that the co-occurrence network structure of the ocular microbiome was altered in the presence of CLOs. In CLO-negative individuals, microbial communities were organized into multiple, distinct subclusters with limited cross-taxa connectivity, indicative of compartmentalized and possibly more stable community interactions. In contrast, CLO-positive networks showed increased complexity, characterized by more edges, larger and more interconnected subclusters, and a higher degree of centrality. While networks cannot infer directionality/causality, and the increased network connectivity observed in CLO-positive microbiomes may partly be attributable to higher species richness—since greater taxonomic diversity increases opportunities for interspecies correlations, this reorganization nonetheless suggests that CLOs and their community of pathogens promote greater connectivity and integration among ocular microbiota, potentially reflecting increased interdependence among taxa or possibly a response to new ecological pressures introduced by CLOs. For example, restructuring of microbial interaction networks in response to specific taxa has been reported in other mucosal ecosystems, such as the gut, oral/dental sites, and the urogenital tract, often associated with shifts in community resilience, nutritional resources, and function (18, 51, 52). Additional studies are needed to further define these interactions over time.

As with any study of this scope, certain limitations should guide interpretation and future research. First, the cross-sectional design precludes causal inference between CLOs and lack of Ct co-infection or trachomatous disease, or how microbiome network interactions evolve over time, although the ethical need to treat active trachoma, Ct, and CLO infections would limit interpretation of longitudinal findings. Second, although beyond the scope of this study, measurements of metabolites and immune responses would be advantageous to provide added insights into our functional pathway findings and hypothesis regarding immunomodulation. Third, low ocular biomass may still contain some contaminants despite rigorous decontamination procedures. Additional studies in other trachoma populations may help to resolve this possibility.

Our findings position CLOs as prevalent and phylogenetically diverse components of the ocular microbiome. Their striking inverse association with Ct and TS/TT suggests that CLOs may influence trachomatous disease through ecological interactions and adaptation, resource competition, and/or immunomodulation, but additional studies are needed to further interrogate these relationships. Both well-known and novel CLO lineages were identified, underscoring the vast, understudied diversity within this phylum, and point to evolutionary relationships relevant for host adaptation. Notably, Unclassified Chlamydiae VI was the largest phylotype and ancestral to *Sorochlamydiaceae*, indicating another novel intermediary link to Chlamydiae families that infect protists (25). At the community level, CLOs are associated with greater microbial evenness and richness and depletion of core functional biosynthetic pathways, suggesting reduced community biosynthetic capacity and niche modification/adaptation. CLO microbiomes were also fundamentally reorganized with increasing network complexity and interconnectivity, which have implications for community stability, function, and further host-microbe interactions. Together, our findings suggest that CLOs not only represent a prevalent and phylogenetically diverse component of the ocular microbiome but underscore the potential for CLOs to influence microbial composition and disease trajectories, highlighting the need to consider intracellular symbionts beyond Ct when investigating ocular health, trachoma pathogenesis, and intervention strategies.

## MATERIALS AND METHODS

### Study design, trachoma grading, and sample collection

The study population comprised 1,059 individuals from 10 villages in Amhara, a trachoma-endemic region of Ethiopia. Participants provided informed consent prior to study enrollment; parents or guardians provided consent for minors.

Trachoma grading was performed as previously described (2). Briefly, upper eyelids were everted, and the tarsal conjunctivae were examined, photographed, and graded using 2.5× ocular loops according to the modified World Health Organization (WHO) grading scale (53): trachomatous inflammation-follicular (TF; >5 follicles >0.5 mm in diameter on the upper tarsal conjunctiva), trachomatous inflammation-intense (TI; >50% of underlying vessels obscured), trachomatous scarring (TS; obvious white scars across tarsal conjunctiva), and trachomatous trichiasis (TT; ≥1 eyelash touching the globe of the eye or evidence of eyelash epilation). We denoted a lack of trachoma signs as T0. Active trachoma was defined by the presence of TF, TI, or both and chronic trachoma by TS, TT, or both. Consensus for the final grade required two of three independent graders who were unaware of demographic and microbiologic data to concur.

After trachoma grading, conjunctival samples were taken from the right and left upper tarsal conjunctivae using FLOQswabs (Copan, Murrieta, CA), and gloves were changed between individuals to avoid cross-contamination. The swabs were placed in 1 mL of M4 collection media (Remel, Lenexa, KA), as previously described (2). All samples were stored at −80°C until use.

### Sample processing, CLO sequencing, and analysis

Genomic (g)DNA was extracted from conjunctival swab samples as previously described using a lysozyme cocktail prior to gDNA purification with the QIAmp DNA Mini Kit (Qiagen, Germantown, MD) (54). The purified gDNA was quantified using Qubit double-stranded DNA (dsDNA) Assay Kit (Invitrogen, Carlsbad, CA) and stored at −20°C until use.

To determine if samples were positive for any chlamydial species, real-time (RT)-qPCR with Chlamydiae phylum-specific primers was employed (55). Positive samples were tested for Ct by *omc*B qPCR (56), which targets the single-copy Ct *omc*B gene. For the detection of CLOs, an ~800 bp fragment of the Chlamydiae phylum-specific region of the 16S rRNA gene was amplified. Data S1 shows primers and thermocycling conditions for these reactions. 16S amplicons were subsequently gel purified for subsequent Sanger sequencing. Reads were aligned against publicly available databases, including NCBI (19) and ChlamDB (21) using BLAST.

All available 16S rRNA sequences for CLOs from NCBI (19), ChlamDB (21), and the ENA (20) in addition to those from this study were subjected to phylogenetic tree construction. A maximum likelihood phylogeny inferred with 1,000 ultrafast bootstraps and 1,000 replicates of the SH-like approximate likelihood ratio test in IQ-TREE was used in the model PMB + F + G4 (IQ-TREE -TESTNEW -bnni -bb 1000 -alrt 1000) (57). Phylotypes were identified using TreeCluster (22).

Associations between CLO status, phylotypes, and categorical metadata variables were evaluated using χ tests, with Fisher’s exact test applied when expected cell counts were below five.

### Metagenomic shotgun sequencing (MSS) and analysis

The processed clinical DNA as described above was subjected to MSS along with controls: (i) nuclease-free water (NTC) processed alongside clinical samples; (ii) unused Copan swabs eluted in buffer and processed as per NTCs; (iii) nuclease-free water and buffer to control for library prep and sequencing contamination; and (iv) ZymoBIOMICS DNA Microbial Community Standards (Zymo Research, Irvine, CA). MSS libraries were constructed using Illumina Nextera XT kits and sequenced using 150 bp paired-end reads on an Illumina Novaseq platform. Processing to remove adaptors and trim read length was performed using Fastp (58). Human reads were removed using Scrubby (59). Post-depletion verification was performed by re-screening the de-hosted reads against the human reference (GRCh38) using Minimap2 (60).

 For microbiome analysis, taxonomic composition of the host-depleted reads was determined using Kraken2 (61). Samples with fewer than 5,000 classified reads were excluded from the analysis to ensure adequate sequencing depth. Given the known lower microbial biomass of conjunctival samples, we used stringent contaminant filtering and negative controls (as described above) to minimize reagent/ambient contaminant taxa, applying the decontam package (62) in R on the Kraken2-derived feature table using both the frequency and prevalence methods, each set at a threshold of 0.3; features flagged as contaminants were removed prior to downstream analyses. Taxa were further filtered to retain only those present in at least 10% of the samples with a minimum of two reads per sample, removing singleton and rare spurious classifications.

To further interrogate the presence/absence of CLOs, host-depleted reads were processed using CZ ID mNGS Illumina pipeline (63). Briefly, non-host reads were aligned to the NCBI nucleotide and protein databases using minimap2 and DIAMOND, respectively, to perform an initial screening for putative short-read alignments. Reads passing this screening step were then assembled into contigs using SPAdes. Reads and contigs were re-aligned using BLAST against custom nucleotide and protein databases composed of reference sequences from taxa identified during the read-level screening, thereby increasing taxonomic specificity.

Alpha diversity was measured using the Shannon Index (i.e., richness and evenness) and observed species counts (i.e., richness). Beta diversity was determined using Bray-Curtis dissimilarity computed in R using the phyloseq package (64). The MaAsLin3 package (26) in R was used to identify differentially abundant species based on CLO presence or absence while adjusting for age, sex, and trachoma grade as covariates with false discovery rate set at 0.05.

Metaphlan2 (65) along with HUManN3 (27) was used to identify functional modules. Functional pathways associated with CLO status, age, sex, and trachoma grade were identified using custom R scripts (Data S2; https://github.com/D-Dean-Lab/CLO_public_scripts).

Species interaction networks were constructed using SparCC correlations (28) implemented in the SpiecEasi R package(66). Prior to network construction, species were filtered to retain those present in at least 10% of samples with at least 10 reads to minimize noise and spurious correlations. SparCC correlations were computed separately for CLO-positive and -negative samples using 20 iterations. Networks were constructed by retaining edges with absolute correlation coefficients ≥ 0.3, capturing both positive (co-occurrence) and negative (mutual exclusion) associations. Communities were detected using the Louvain algorithm (25), and subclusters were matched between CLO-positive and -negative networks.

## Data Availability

The FASTQ files for all the metagenomes were submitted to the NCBI-SRA under BioProject accession number PRJNA1440961. All scripts generated for this research relating to data visualization and statistical analyses can be found at https://github.com/D-Dean-Lab/CLO_public_scripts.

## SUPPLEMENTAL MATERIAL

Supplemental Figures mbio.00534-26-s0001.pdf Figures S1–S3; captions for Tables S1–S4; Data S1 and S2.

Table S1 mbio.00534-26-s0002.xlsx Association of CLO phylotypes with age, sex, trachoma grade, and Ct infection status.

Table S2 mbio.00534-26-s0003.xlsx Differentially prevalent microbial species between CLO+ and CLO− ocular microbiome samples identified by MaAsLin3.

Table S3 mbio.00534-26-s0004.xlsx Species-level contributions to CLO-associated functional pathway.

Table S4 mbio.00534-26-s0005.xlsx Species network properties from SparCC co-occurrence analysis.

ASM does not own the copyrights to Supplemental Material that may be linked to, or accessed through, an article. The authors have granted ASM a non-exclusive, world-wide license to publish the Supplemental Material files. Please contact the corresponding author directly for reuse.
