# Supplementary material for: Phylogenetic diversity, functional pathways, and network interactions of ocular chlamydia-like organisms (CLOs) in trachoma-endemic Ethiopia
Source: mBio. 2026 Apr 23;17(5):e00534-26. doi: 10.1128/mbio.00534-26 (PMC13170232; doi:10.1128/mbio.00534-26)
Supplement: Supplemental Figures — Figures S1–S3; captions for Tables S1–S4; Data S1 and S2. [file mbio.00534-26-s0001.pdf]

Phylogenetic Diversity, Functional Pathways, and Network Interactions of Ocular Chlamydia-Like Organisms (CLOs) in Trachoma-Endemic Ethiopia

Olusola Olagoke,[a,b] Xinhe Zheng,[a] Seongwon Chung,[a] Hiwot Degineh Mengistie,[c] Kaleb Asfaha,[a,d] Timothy D. Read,[e] and Deborah Dean[a,b,f,g#]

[a]Department of Pediatrics, Division of Global Health and Infectious Diseases, University of California San Francisco School of Medicine, San Francisco, CA, USA
[b]Benioff Center for Microbiome Medicine, University of California San Francisco, CA, USA
[c]Bahir Dar Specialty Eye Center, Bahir Dar, Ethiopia
[d]School of Optometry & Vision Science, University of California Berkeley, Berkeley, CA, USA
[e]Department of Medicine, Division of Infectious Diseases, Emory University School of Medicine, Atlanta, GA, USA
[f]Department of Medicine, Division of Global Health and Infectious Diseases, University of California San Francisco School of Medicine, San Francisco, CA, USA
[g]Department of Bioengineering, Joint Program, University of California San Francisco and University of California Berkeley, San Francisco, CA, USA

SUPPLEMENTARY MATERIAL

Figures:

Figure S1. Alpha diversity of microbial communities stratified by CLO status and demographic and clinical variables. Observed species richness is shown stratified by S1A) age group, S1B) sex, and S1C) trachoma grade. Shannon diversity index is similarly stratified by S1D) age group, S1E) sex, and S1F) trachoma grade. Data points represent individual samples, colored by CLO status (red = CLO-positive, blue = CLO-negative). Comparisons were performed using the Wilcoxon rank-sum test, with p values indicated above each comparison. Faceting displays results stratified by age group, sex, and trachoma grade. Only comparisons with a significant p value (<0.05) are noted.

Figure S2: CLO-associated differences in ocular microbiome community composition. Principal coordinates analysis (PCoA) based on Bray–Curtis dissimilarities comparing ocular microbiome composition between CLO-positive (red) and CLO-negative (blue) individuals. Analyses were restricted to *C. trachomatis*–negative participants to avoid confounding by Ct infection. Panels show stratified comparisons across age groups (children, adolescents, adults and older adults), sex (male and female), and trachoma grades (T0, TF/TI, TS/TT). Each point represents an individual sample, and ellipses denote 95% confidence intervals. The percentage of variance explained by each principal coordinate is indicated on the axes. Differences in community composition between groups were assessed using permutational multivariate analysis of variance (PERMANOVA), with corresponding p values and R^2 statistics shown within each panel.

Figure S3. CLO-associated functional pathway differences in ocular microbiomes. Differential pathway abundance between CLO-positive and CLO-negative ocular microbiomes was assessed using HUMAnN-derived community-level functional profiles. The plot shows pathways with statistically significant differences (FDR-adjusted $p<0.05$) in the indicated subgroup: S3A) Children, S3B) Females, S3C) Males, S3D) TS/TT, and S3E) Older adults. Only analyses yielding

more than one differential pathway are displayed. Pathways are grouped by functional category and colored accordingly (legend, right). Dot size is proportional to the magnitude of the effect size.

Data:

Supplementary Data S1. Primers and PCR conditions for the Chlamydiae-specific 16S touchdown PCR.
Supplementary Data S2. Community level pathways R script.

Tables:

Table S1: Association of CLO phylotypes with age, sex, trachoma grade and Ct infection status.
Table S2. Differentially abundant and prevalent microbial species between CLO-positive and CLO-negative ocular microbiome samples identified by MaAsLin3.
Table S3a-g: Species-level contributions to CLO-associated functional pathway in the ocular microbiome of children
Table S4. Species network properties from SparCC co-occurrence analysis in CLO-positive and CLO-negative ocular microbiome samples.

S1A

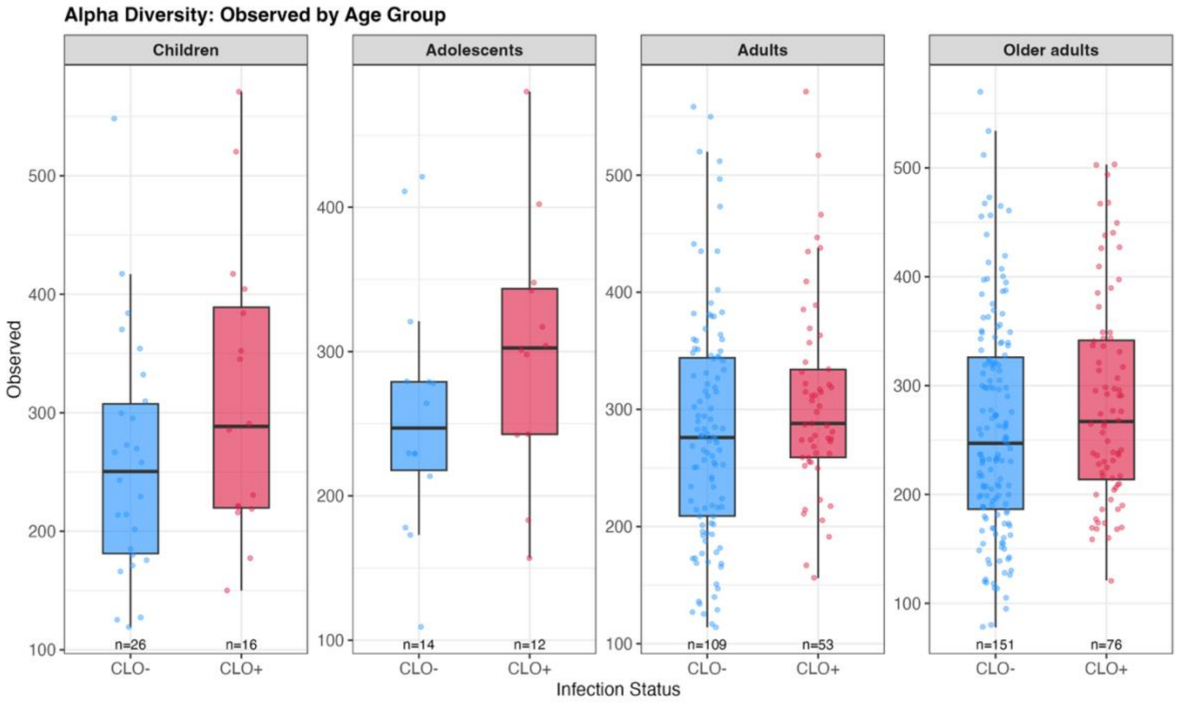

Alpha Diversity: Observed by Age Group
Children
Adolescents
Adults
Older adults
Observed
n=26
n=16
n=14
n=12
n=109
n=53
n=151
n=76
CLO-
CLO+
Infection Status

S1B

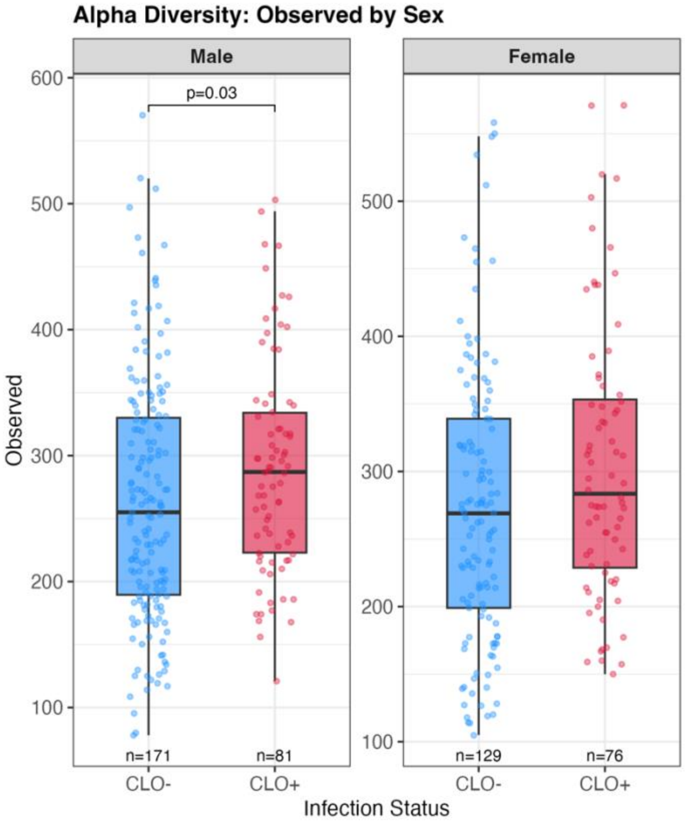

Alpha Diversity: Observed by Sex
Male
Female
p=0.03
Observed
n=171
n=81
n=129
n=76
CLO-
CLO+
Infection Status

S1C

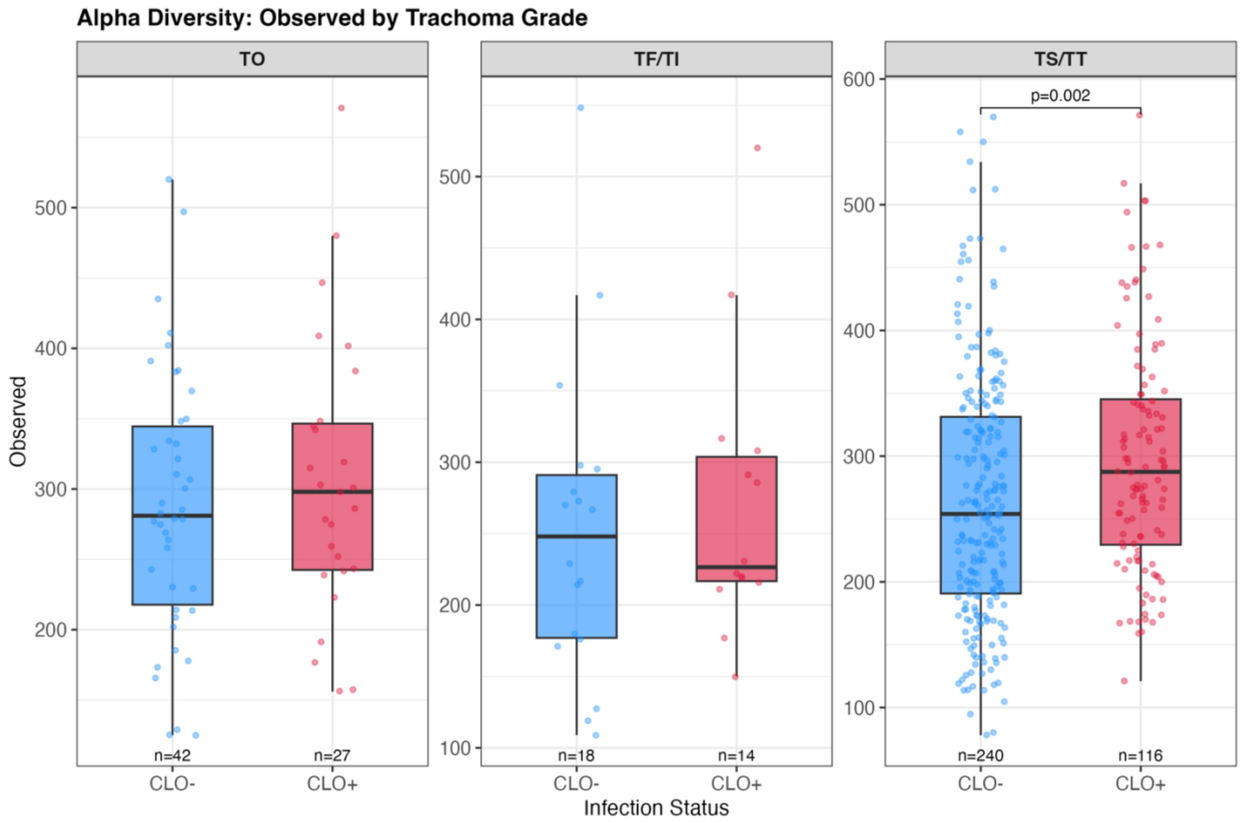
Alpha Diversity: Observed by Trachoma Grade
TO
TF/TI
TS/TT
p=0.002
Observed
n=42
n=27
n=18
n=14
n=240
n=116
CLO-
CLO+
Infection Status

S1D

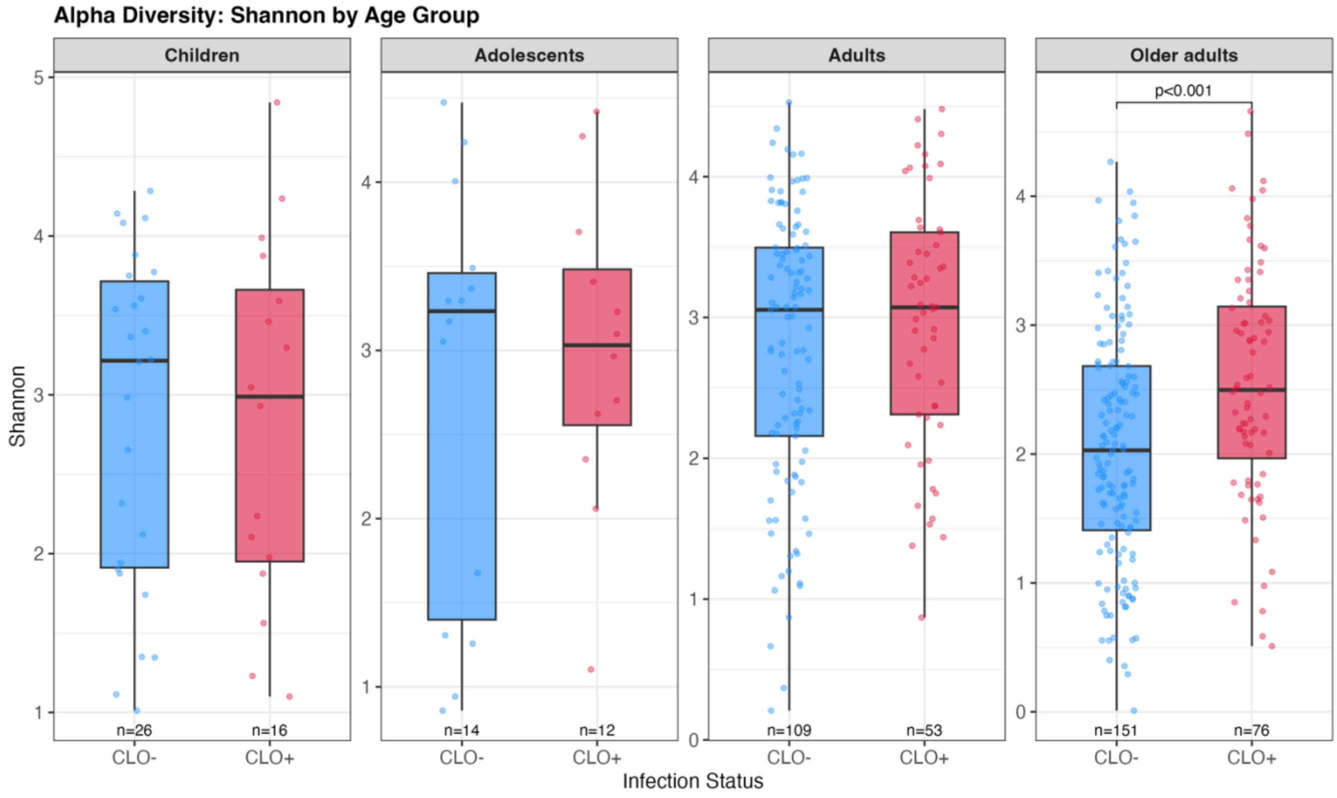
Alpha Diversity: Shannon by Age Group
Children
Adolescents
Adults
Older adults
p<0.001
Shannon
n=26
n=16
n=14
n=12
n=109
n=53
n=151
n=76
CLO-
CLO+
Infection Status

S1E

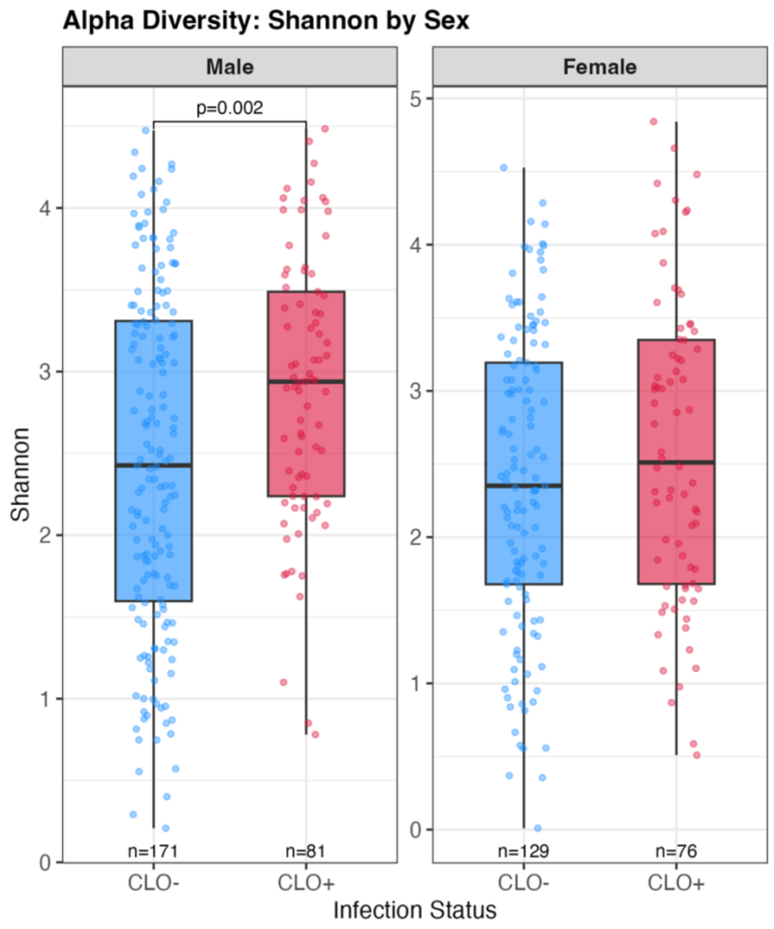

Alpha Diversity: Shannon by Sex
Male
Female
p=0.002
Shannon
0
1
2
3
4
5
n=171
n=81
n=129
n=76
CLO-
CLO+
CLO-
CLO+
Infection Status

S1F

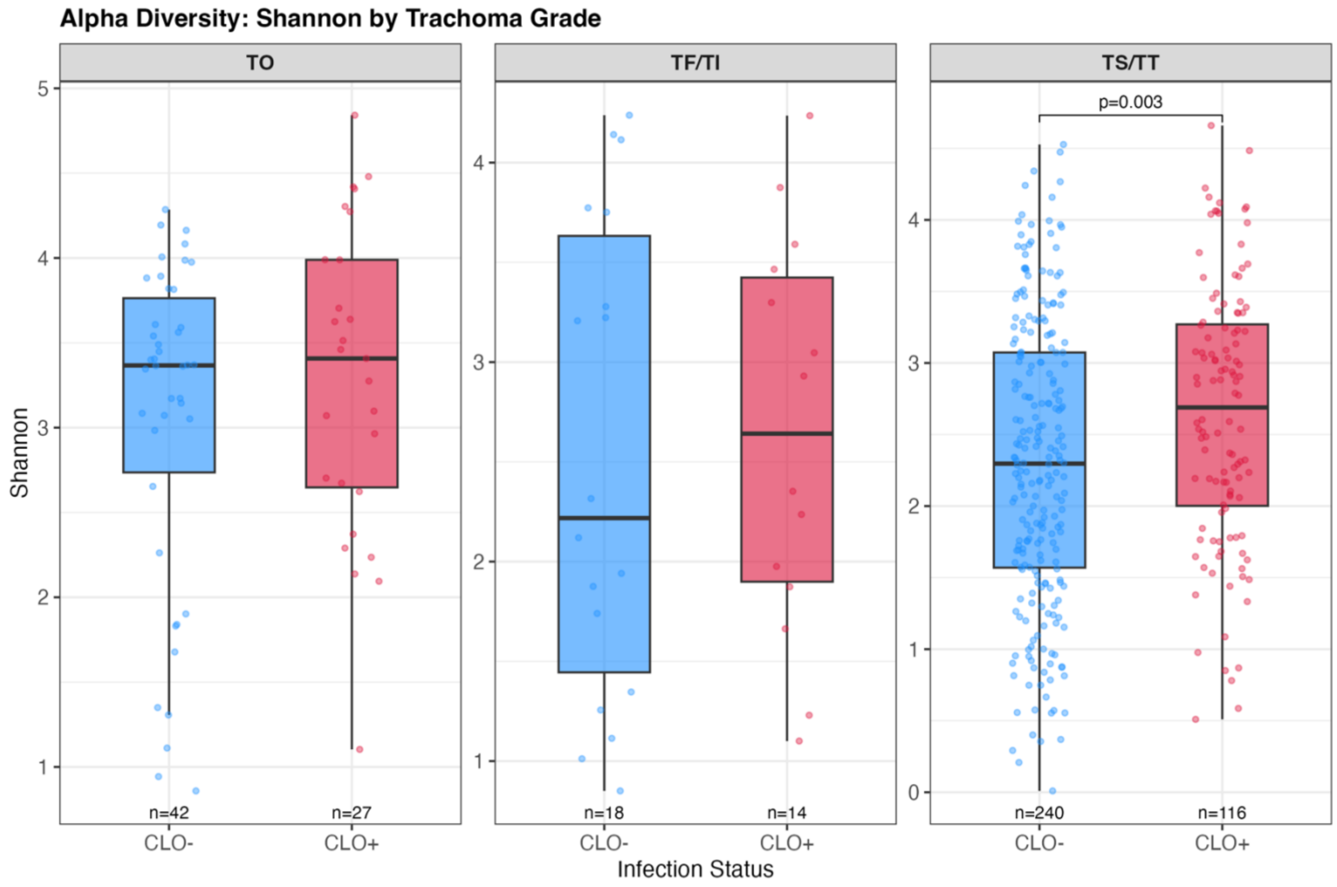

Alpha Diversity: Shannon by Trachoma Grade
TO
TF/TI
TS/TT
p=0.003
Shannon
1
2
3
4
5
0
n=42
n=27
n=18
n=14
n=240
n=116
CLO-
CLO+
CLO-
CLO+
CLO-
CLO+
Infection Status

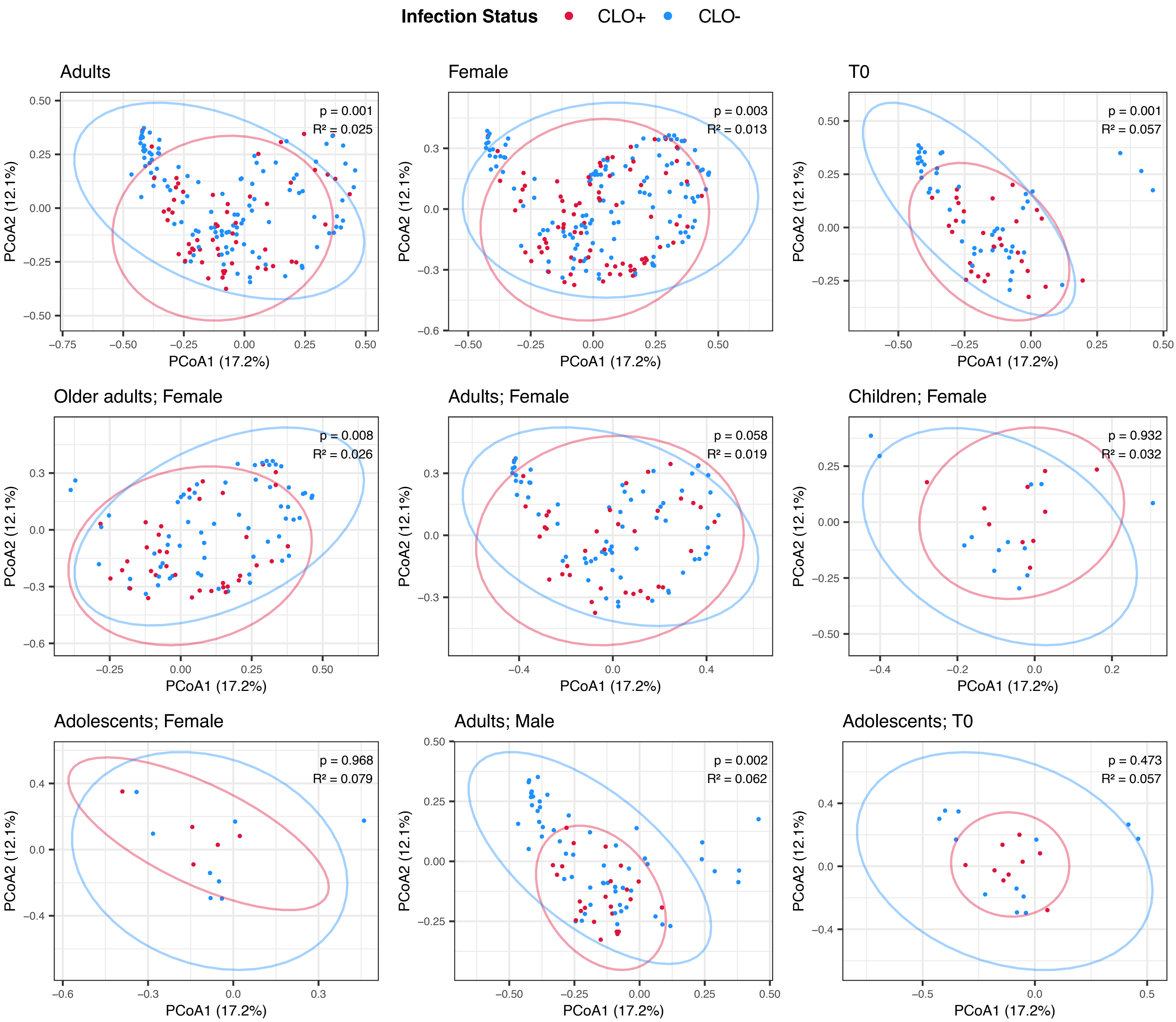

Infection Status
CLO+
CLO-
Adults
p = 0.001
R² = 0.025
Female
p = 0.003
R² = 0.013
T0
p = 0.001
R² = 0.057
Older adults; Female
p = 0.008
R² = 0.026
Adults; Female
p = 0.058
R² = 0.019
Children; Female
p = 0.932
R² = 0.032
Adolescents; Female
p = 0.968
R² = 0.079
Adults; Male
p = 0.002
R² = 0.062
Adolescents; T0
p = 0.473
R² = 0.057
PCoA1 (17.2%)
PCoA2 (12.1%)

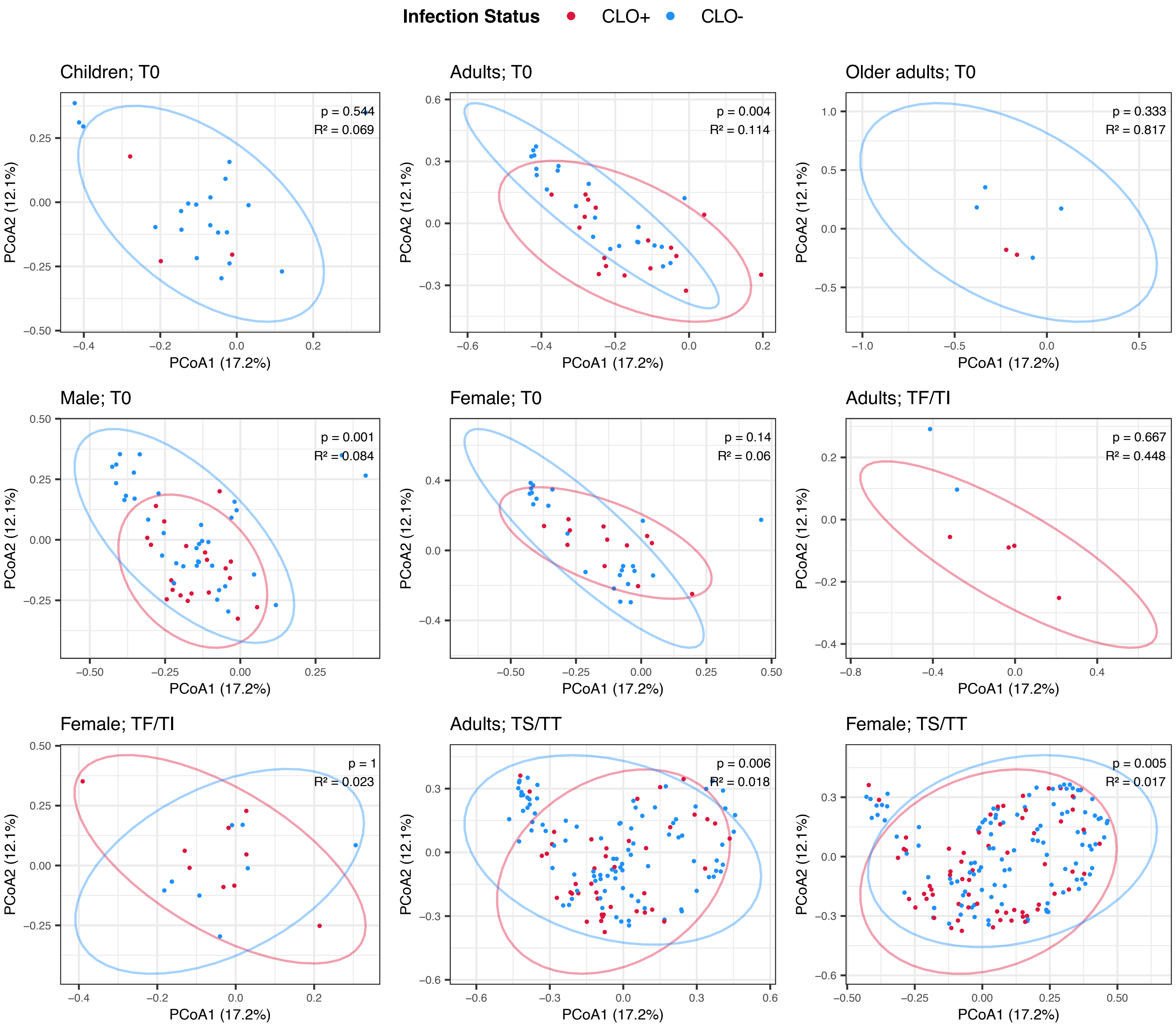
Infection Status CLO+ CLO-
Children; T0
p = 0.544
R² = 0.069
Adults; T0
p = 0.004
R² = 0.114
Older adults; T0
p = 0.333
R² = 0.817
Male; T0
p = 0.001
R² = 0.084
Female; T0
p = 0.14
R² = 0.06
Adults; TF/TI
p = 0.667
R² = 0.448
Female; TF/TI
p = 1
R² = 0.023
Adults; TS/TT
p = 0.006
R² = 0.018
Female; TS/TT
p = 0.005
R² = 0.017
PCoA1 (17.2%)
PCoA2 (12.1%)

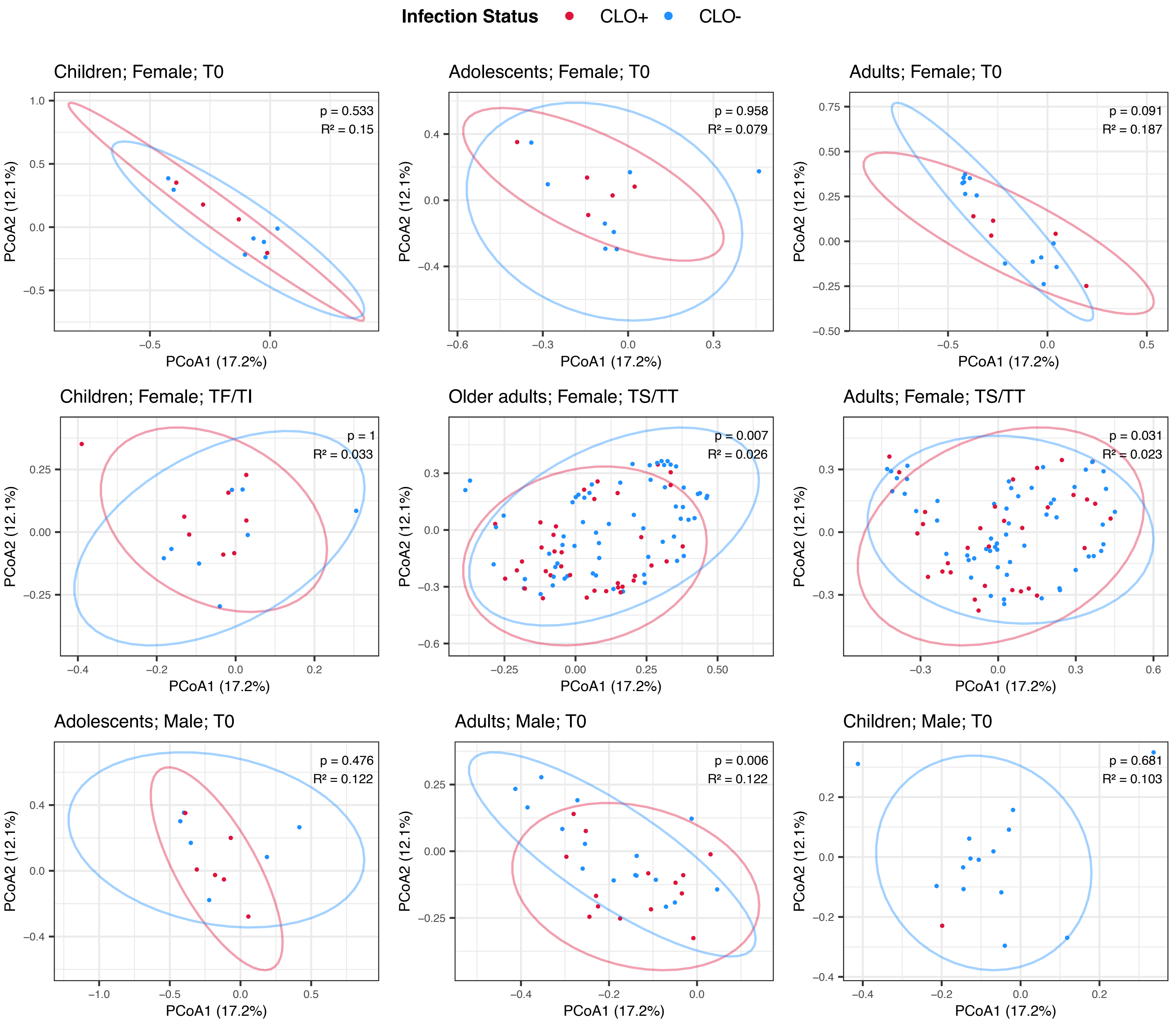

Infection Status
CLO+
CLO-
Children; Female; T0
p = 0.533
R² = 0.15
Adolescents; Female; T0
p = 0.958
R² = 0.079
Adults; Female; T0
p = 0.091
R² = 0.187
Children; Female; TF/TI
p = 1
R² = 0.033
Older adults; Female; TS/TT
p = 0.007
R² = 0.026
Adults; Female; TS/TT
p = 0.031
R² = 0.023
Adolescents; Male; T0
p = 0.476
R² = 0.122
Adults; Male; T0
p = 0.006
R² = 0.122
Children; Male; T0
p = 0.681
R² = 0.103
PCoA1 (17.2%)
PCoA2 (12.1%)

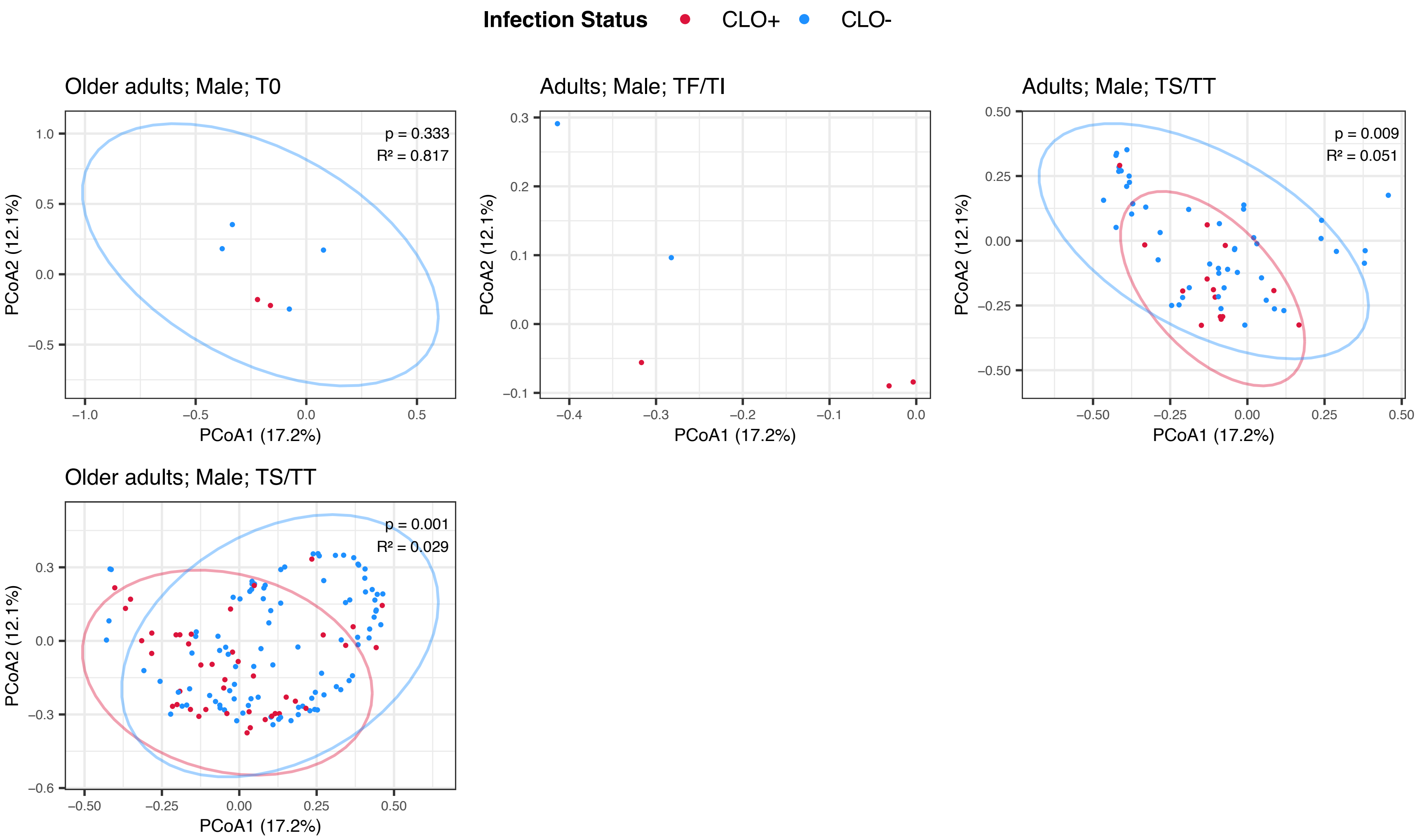

Infection Status
CLO+
CLO-
Older adults; Male; T0
p = 0.333
R² = 0.817
Adults; Male; TF/TI
Adults; Male; TS/TT
p = 0.009
R² = 0.051
Older adults; Male; TS/TT
p = 0.001
R² = 0.029
PCoA1 (17.2%)
PCoA2 (12.1%)

S3A

Children

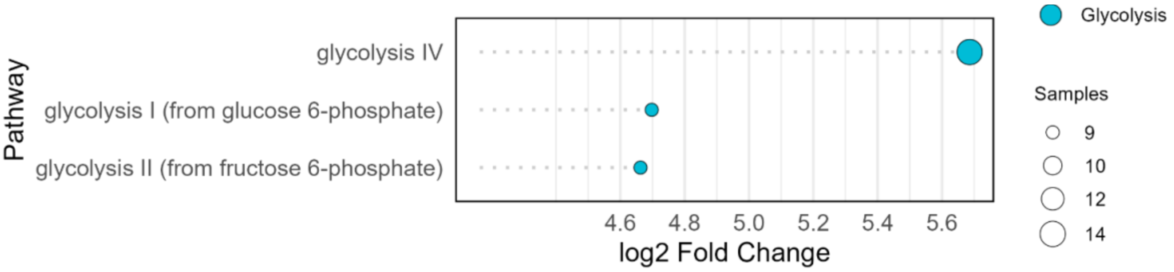

S3B

Females

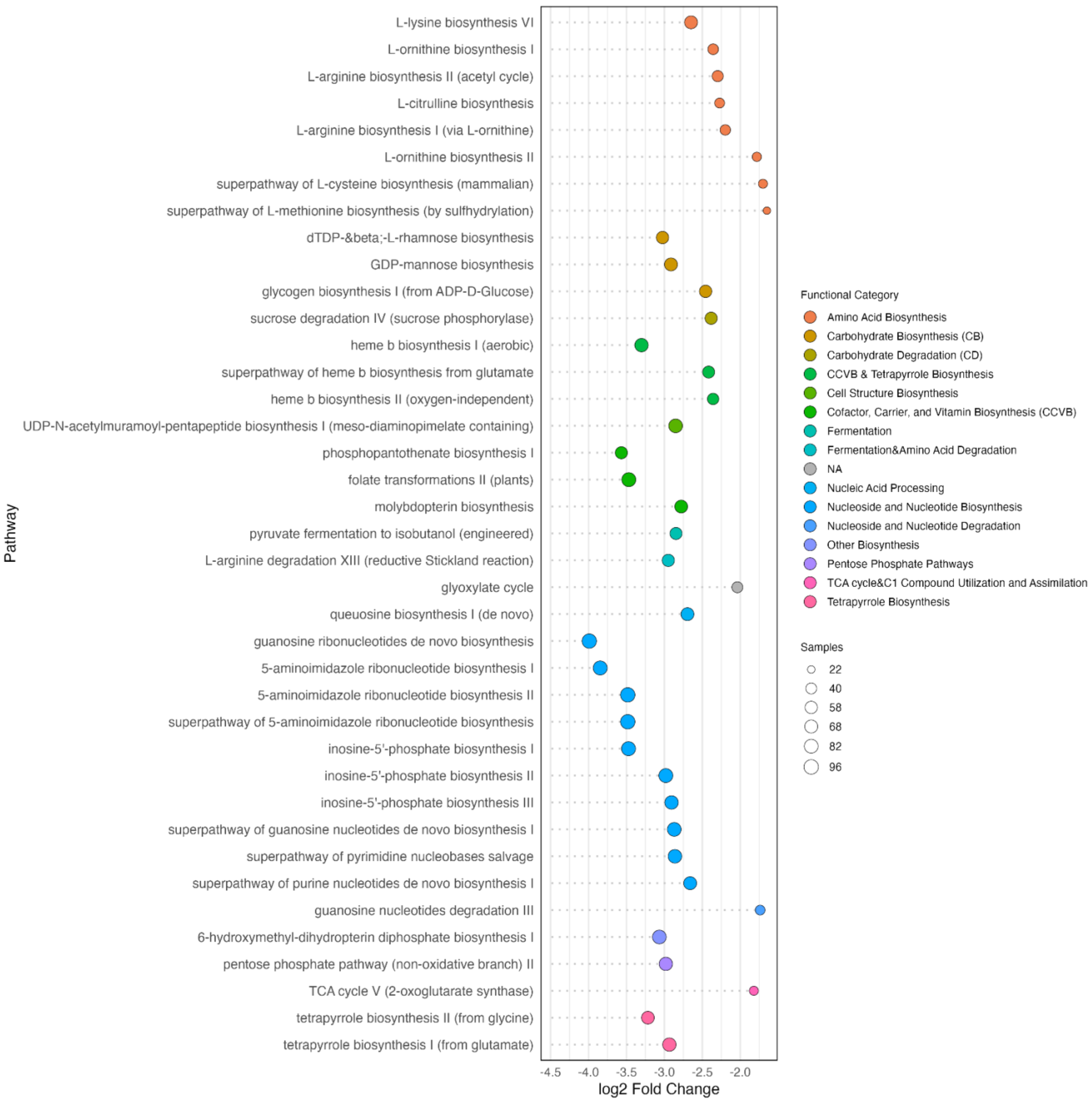

S3C

Males

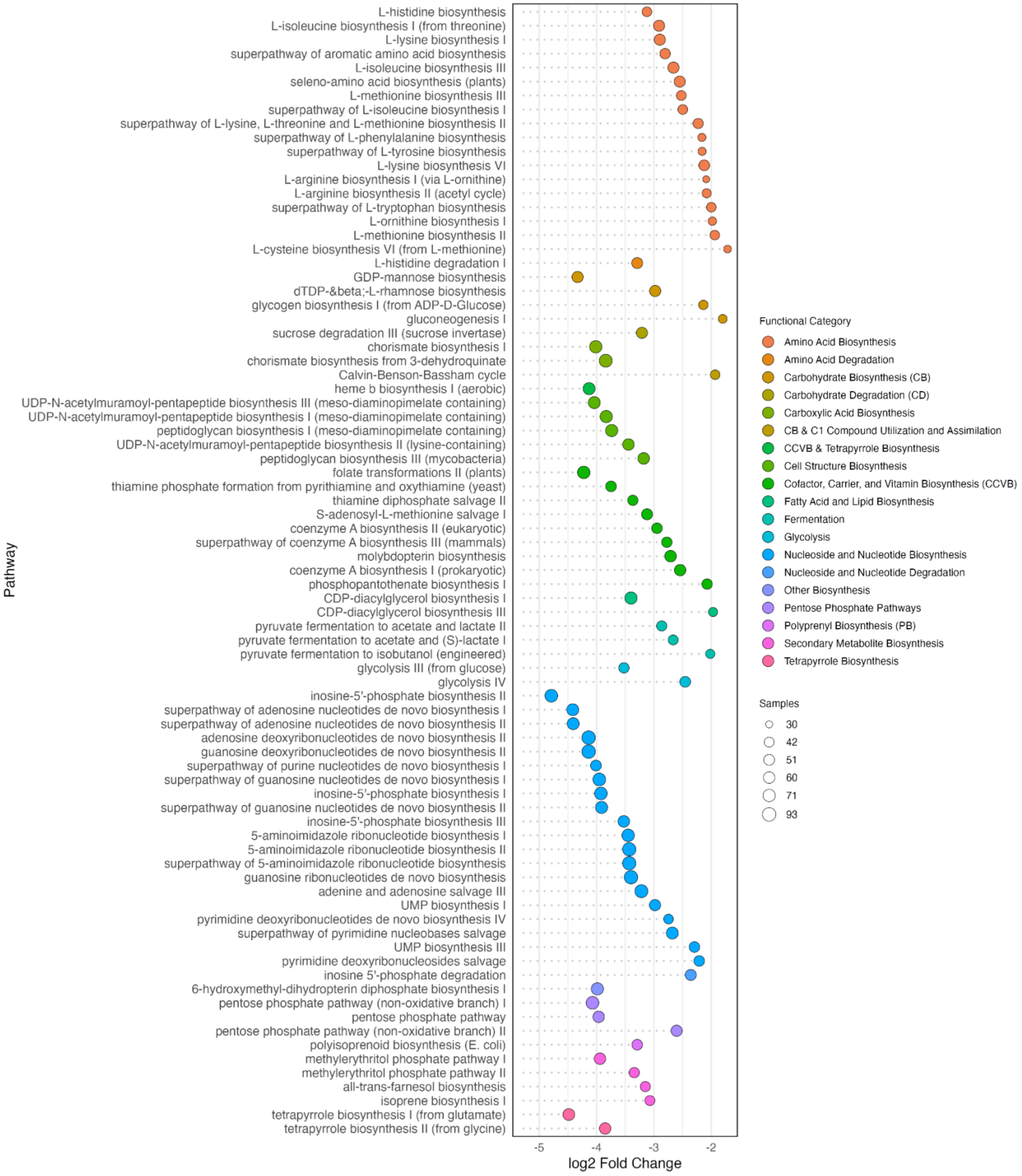
L-histidine biosynthesis
L-isoleucine biosynthesis I (from threonine)
L-lysine biosynthesis I
superpathway of aromatic amino acid biosynthesis
L-isoleucine biosynthesis III
seleno-amino acid biosynthesis (plants)
L-methionine biosynthesis III
superpathway of L-isoleucine biosynthesis I
superpathway of L-lysine, L-threonine and L-methionine biosynthesis II
superpathway of L-phenylalanine biosynthesis
superpathway of L-tyrosine biosynthesis
L-lysine biosynthesis VI
L-arginine biosynthesis I (via L-ornithine)
L-arginine biosynthesis II (acetyl cycle)
superpathway of L-tryptophan biosynthesis
L-ornithine biosynthesis I
L-methionine biosynthesis II
L-cysteine biosynthesis VI (from L-methionine)
L-histidine degradation I
GDP-mannose biosynthesis
dTDP-β-L-rhamnose biosynthesis
glycogen biosynthesis I (from ADP-D-Glucose)
gluconeogenesis I
sucrose degradation III (sucrose invertase)
chorismate biosynthesis I
chorismate biosynthesis from 3-dehydroquinate
Calvin-Benson-Bassham cycle
heme b biosynthesis I (aerobic)
UDP-N-acetylmuramoyl-pentapeptide biosynthesis III (meso-diaminopimelate containing)
UDP-N-acetylmuramoyl-pentapeptide biosynthesis I (meso-diaminopimelate containing)
peptidoglycan biosynthesis I (meso-diaminopimelate containing)
UDP-N-acetylmuramoyl-pentapeptide biosynthesis II (lysine-containing)
peptidoglycan biosynthesis III (mycobacteria)
folate transformations II (plants)
thiamine phosphate formation from pyrithiamine and oxythiamine (yeast)
thiamine diphosphate salvage II
S-adenosyl-L-methionine salvage I
coenzyme A biosynthesis II (eukaryotic)
superpathway of coenzyme A biosynthesis III (mammals)
molybdopterin biosynthesis
coenzyme A biosynthesis I (prokaryotic)
phosphopantothenate biosynthesis I
CDP-diacylglycerol biosynthesis I
CDP-diacylglycerol biosynthesis III
pyruvate fermentation to acetate and lactate II
pyruvate fermentation to acetate and (S)-lactate I
pyruvate fermentation to isobutanol (engineered)
glycolysis III (from glucose)
glycolysis IV
inosine-5'-phosphate biosynthesis II
superpathway of adenosine nucleotides de novo biosynthesis I
superpathway of adenosine nucleotides de novo biosynthesis II
adenosine deoxyribonucleotides de novo biosynthesis II
guanosine deoxyribonucleotides de novo biosynthesis II
superpathway of purine nucleotides de novo biosynthesis I
superpathway of guanosine nucleotides de novo biosynthesis I
inosine-5'-phosphate biosynthesis I
superpathway of guanosine nucleotides de novo biosynthesis II
inosine-5'-phosphate biosynthesis III
5-aminoimidazole ribonucleotide biosynthesis I
5-aminoimidazole ribonucleotide biosynthesis II
superpathway of 5-aminoimidazole ribonucleotide biosynthesis
guanosine ribonucleotides de novo biosynthesis
adenine and adenosine salvage III
UMP biosynthesis I
pyrimidine deoxyribonucleotides de novo biosynthesis IV
superpathway of pyrimidine nucleobases salvage
UMP biosynthesis III
pyrimidine deoxyribonucleosides salvage
inosine 5'-phosphate degradation
6-hydroxymethyl-dihydropterin diphosphate biosynthesis I
pentose phosphate pathway (non-oxidative branch) I
pentose phosphate pathway
pentose phosphate pathway (non-oxidative branch) II
polyisoprenoid biosynthesis (E. coli)
methylerythritol phosphate pathway I
methylerythritol phosphate pathway II
all-trans-farnesol biosynthesis
isoprene biosynthesis I
tetrapyrrole biosynthesis I (from glutamate)
tetrapyrrole biosynthesis II (from glycine)
Pathway
-5
-4
-3
-2
log2 Fold Change
Functional Category
Amino Acid Biosynthesis
Amino Acid Degradation
Carbohydrate Biosynthesis (CB)
Carbohydrate Degradation (CD)
Carboxylic Acid Biosynthesis
CB & C1 Compound Utilization and Assimilation
CCVB & Tetrapyrrole Biosynthesis
Cell Structure Biosynthesis
Cofactor, Carrier, and Vitamin Biosynthesis (CCVB)
Fatty Acid and Lipid Biosynthesis
Fermentation
Glycolysis
Nucleoside and Nucleotide Biosynthesis
Nucleoside and Nucleotide Degradation
Other Biosynthesis
Pentose Phosphate Pathways
Polyprenyl Biosynthesis (PB)
Secondary Metabolite Biosynthesis
Tetrapyrrole Biosynthesis
Samples
30
42
51
60
71
93

S3D

TS/TT

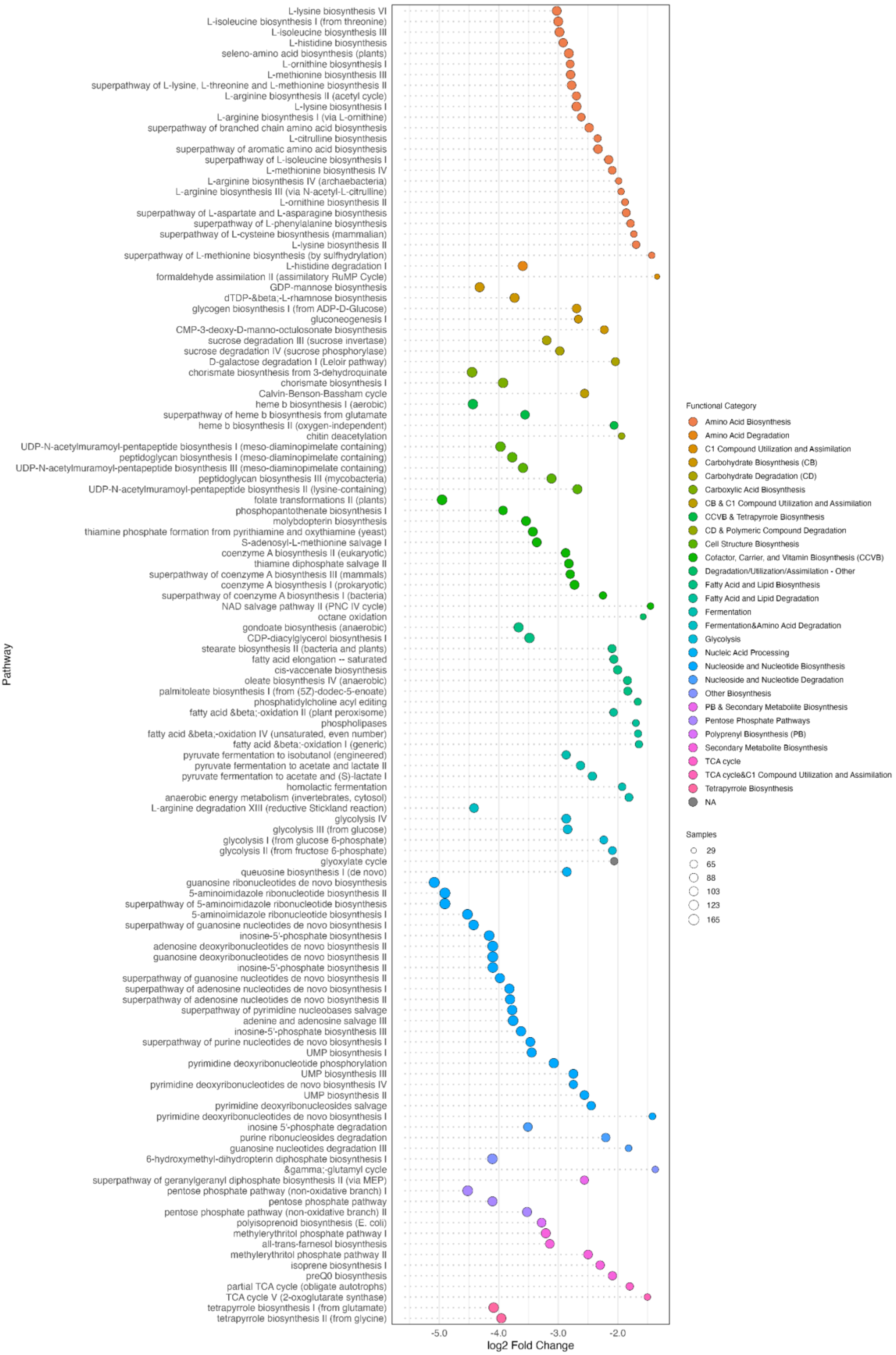

L-lysine biosynthesis VI
L-isoleucine biosynthesis I (from threonine)
L-isoleucine biosynthesis III
L-histidine biosynthesis
seleno-amino acid biosynthesis (plants)
L-ornithine biosynthesis I
L-methionine biosynthesis III
superpathway of L-lysine, L-threonine and L-methionine biosynthesis II
L-arginine biosynthesis II (acetyl cycle)
L-lysine biosynthesis I
L-arginine biosynthesis I (via L-ornithine)
superpathway of branched chain amino acid biosynthesis
L-citrulline biosynthesis
superpathway of aromatic amino acid biosynthesis
superpathway of L-isoleucine biosynthesis I
L-methionine biosynthesis IV
L-arginine biosynthesis IV (archaebacteria)
L-arginine biosynthesis III (via N-acetyl-L-citrulline)
L-ornithine biosynthesis II
superpathway of L-aspartate and L-asparagine biosynthesis
superpathway of L-phenylalanine biosynthesis
superpathway of L-cysteine biosynthesis (mammalian)
L-lysine biosynthesis II
superpathway of L-methionine biosynthesis (by sulfhydrylation)
L-histidine degradation I
formaldehyde assimilation II (assimilatory RuMP Cycle)
GDP-mannose biosynthesis
dTDP-β-L-rhamnose biosynthesis
glycogen biosynthesis I (from ADP-D-Glucose)
gluconeogenesis I
CMP-3-deoxy-D-manno-octulosonate biosynthesis
sucrose degradation III (sucrose invertase)
sucrose degradation IV (sucrose phosphorylase)
D-galactose degradation I (Leloir pathway)
chorismate biosynthesis from 3-dehydroquinate
chorismate biosynthesis I
Calvin-Benson-Bassham cycle
heme b biosynthesis I (aerobic)
superpathway of heme b biosynthesis from glutamate
heme b biosynthesis II (oxygen-independent)
chitin deacetylation
UDP-N-acetylmuramoyl-pentapeptide biosynthesis I (meso-diaminopimelate containing)
peptidoglycan biosynthesis I (meso-diaminopimelate containing)
UDP-N-acetylmuramoyl-pentapeptide biosynthesis III (meso-diaminopimelate containing)
peptidoglycan biosynthesis III (mycobacteria)
UDP-N-acetylmuramoyl-pentapeptide biosynthesis II (lysine-containing)
folate transformations II (plants)
phosphopantothenate biosynthesis I
molybdopterin biosynthesis
thiamine phosphate formation from pyrithiamine and oxythiamine (yeast)
S-adenosyl-L-methionine salvage I
coenzyme A biosynthesis II (eukaryotic)
thiamine diphosphate salvage II
superpathway of coenzyme A biosynthesis III (mammals)
coenzyme A biosynthesis I (prokaryotic)
superpathway of coenzyme A biosynthesis I (bacteria)
NAD salvage pathway II (PNC IV cycle)
octane oxidation
gondoate biosynthesis (anaerobic)
CDP-diacylglycerol biosynthesis I
stearate biosynthesis II (bacteria and plants)
fatty acid elongation -- saturated
cis-vaccenate biosynthesis
oleate biosynthesis IV (anaerobic)
palmitoleate biosynthesis I (from (5Z)-dodec-5-enoate)
phosphatidylcholine acyl editing
fatty acid β-oxidation II (plant peroxisome)
phospholipases
fatty acid β-oxidation IV (unsaturated, even number)
fatty acid β-oxidation I (generic)
pyruvate fermentation to isobutanol (engineered)
pyruvate fermentation to acetate and lactate II
pyruvate fermentation to acetate and (S)-lactate I
homolactic fermentation
anaerobic energy metabolism (invertebrates, cytosol)
L-arginine degradation XIII (reductive Stickland reaction)
glycolysis IV
glycolysis III (from glucose)
glycolysis I (from glucose 6-phosphate)
glycolysis II (from fructose 6-phosphate)
glyoxylate cycle
queuosine biosynthesis I (de novo)
guanosine ribonucleotides de novo biosynthesis
5-aminoimidazole ribonucleotide biosynthesis II
superpathway of 5-aminoimidazole ribonucleotide biosynthesis
5-aminoimidazole ribonucleotide biosynthesis I
superpathway of guanosine nucleotides de novo biosynthesis I
inosine-5'-phosphate biosynthesis I
adenosine deoxyribonucleotides de novo biosynthesis II
guanosine deoxyribonucleotides de novo biosynthesis II
inosine-5'-phosphate biosynthesis II
superpathway of guanosine nucleotides de novo biosynthesis II
superpathway of adenosine nucleotides de novo biosynthesis I
superpathway of adenosine nucleotides de novo biosynthesis II
superpathway of pyrimidine nucleobases salvage
adenine and adenosine salvage III
inosine-5'-phosphate biosynthesis III
superpathway of purine nucleotides de novo biosynthesis I
UMP biosynthesis I
pyrimidine deoxyribonucleotide phosphorylation
UMP biosynthesis III
pyrimidine deoxyribonucleotides de novo biosynthesis IV
UMP biosynthesis II
pyrimidine deoxyribonucleosides salvage
pyrimidine deoxyribonucleotides de novo biosynthesis I
inosine 5'-phosphate degradation
purine ribonucleosides degradation
guanosine nucleotides degradation III
6-hydroxymethyl-dihydropterin diphosphate biosynthesis I
γ-glutamyl cycle
superpathway of geranylgeranyl diphosphate biosynthesis II (via MEP)
pentose phosphate pathway (non-oxidative branch) I
pentose phosphate pathway
pentose phosphate pathway (non-oxidative branch) II
polyisoprenoid biosynthesis (E. coli)
methylerythritol phosphate pathway I
all-trans-farnesol biosynthesis
methylerythritol phosphate pathway II
isoprene biosynthesis I
preQ0 biosynthesis
partial TCA cycle (obligate autotrophs)
TCA cycle V (2-oxoglutarate synthase)
tetrapyrrole biosynthesis I (from glutamate)
tetrapyrrole biosynthesis II (from glycine)
Pathway
-5.0
-4.0
-3.0
-2.0
log2 Fold Change
Functional Category
Amino Acid Biosynthesis
Amino Acid Degradation
C1 Compound Utilization and Assimilation
Carbohydrate Biosynthesis (CB)
Carbohydrate Degradation (CD)
Carboxylic Acid Biosynthesis
CB & C1 Compound Utilization and Assimilation
CCVB & Tetrapyrrole Biosynthesis
CD & Polymeric Compound Degradation
Cell Structure Biosynthesis
Cofactor, Carrier, and Vitamin Biosynthesis (CCVB)
Degradation/Utilization/Assimilation - Other
Fatty Acid and Lipid Biosynthesis
Fatty Acid and Lipid Degradation
Fermentation
Fermentation&Amino Acid Degradation
Glycolysis
Nucleic Acid Processing
Nucleoside and Nucleotide Biosynthesis
Nucleoside and Nucleotide Degradation
Other Biosynthesis
PB & Secondary Metabolite Biosynthesis
Pentose Phosphate Pathways
Polyprenyl Biosynthesis (PB)
Secondary Metabolite Biosynthesis
TCA cycle
TCA cycle&C1 Compound Utilization and Assimilation
Tetrapyrrole Biosynthesis
NA
Samples
29
65
88
103
123
165

S3E

<u>Older Adults</u>

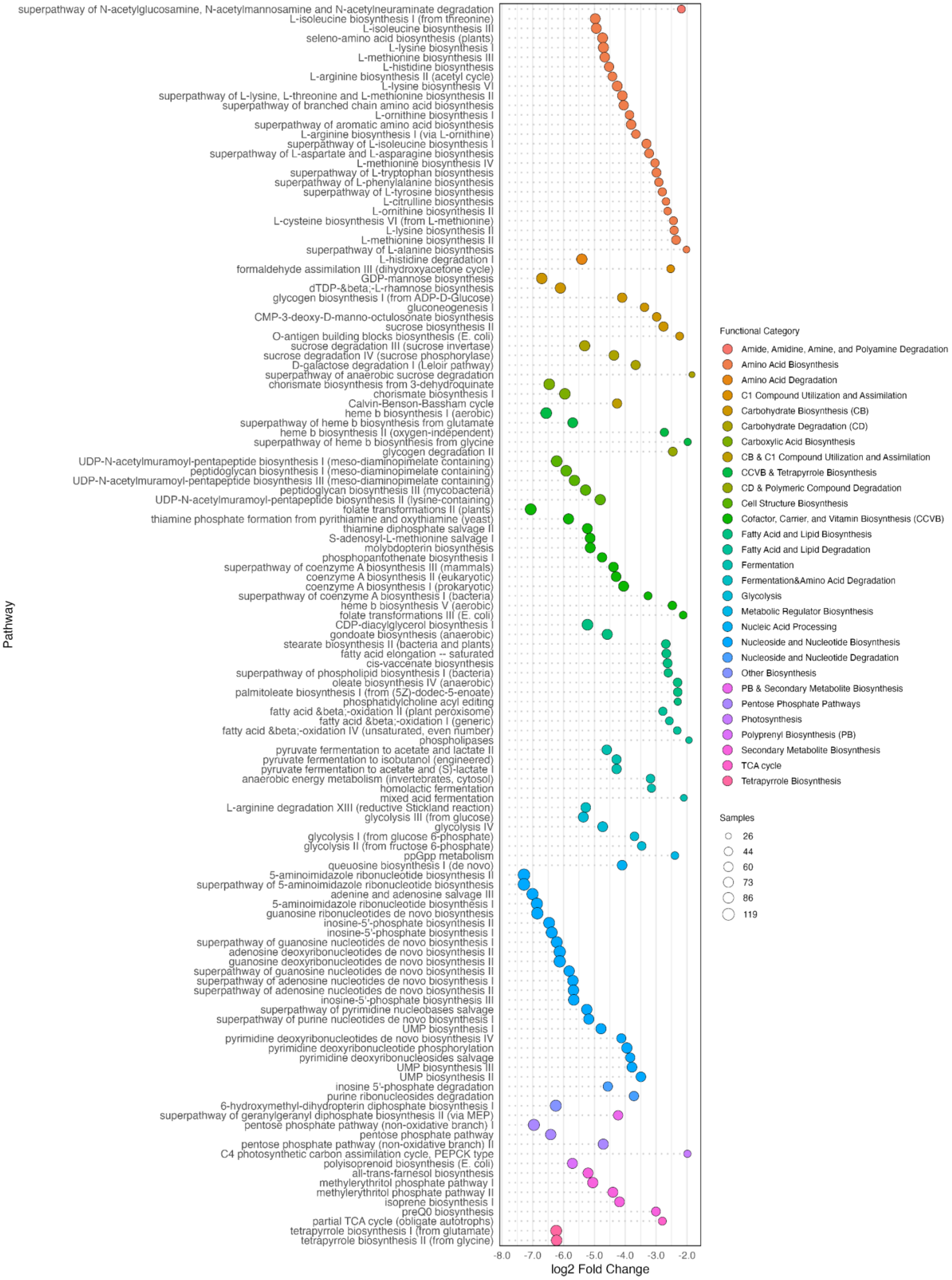

Supplementary Data S1. Primers and PCR conditions for the Chlamydiae-specific 16S touchdown PCR

S1A: Primers

Primer name	Type	Sequence (5'→3')
16S-F	Forward	CGGCGTGGATGAGGCAT
16S-R	Reverse	TAGGCGGAAAGGTAAGTTAGT

S1B: Reaction composition (per 28 µL reaction)

Component	Volume (µL)
AmpliTaq™ 360 Master Mix (2×)	12.5
Nuclease-free H_2O	9.0
Primer 16S-IGF (forward)	0.75
Primer 16S-R (reverse)	0.75
DNA template	5.0
Total	28.0

S1C: Cycling program (touchdown then standard)

Step	Temperature (°C)	Time	Cycles	Notes
Initial denaturation	95	10 min	1	
Touchdown block A	95 / 61 / 72	30s/45s/45s	2	3-step cycles; anneal at 61 °C
Touchdown block B	95 / 60 / 72	30s/45s/45s	2	anneal at 60 °C
Touchdown block C	95 / 59 / 72	30s/45s/45s	2	anneal at 59 °C
Touchdown block D	95 / 58 / 72	30s/45s/45s	2	anneal at 58 °C
Touchdown block E	95 / 57 / 72	30s/45s/45s	2	anneal at 57 °C
Touchdown block F	95 / 56.2 / 72	30s/45s/45s	2	anneal at 56.2 °C
Amplification	95 / 55 / 72	30s/45s/45s	30	standard 3-step PCR; anneal at 55 °C
Final extension	72	7 min	1	
Hold	4	∞	1	

Notes. Each cycle is a 3-step PCR (denaturation / annealing / extension).

Supplementary Data S2. Summary Level Pathways.

```
# ===========================================================
#
# Purpose:
#   - Load HUMAnN (or similar) pathway abundance wide table + metadata
#   - Remove species-resolved rows (keep community-level pathways)
#   - Long-format join to metadata (optionally filtering samples)
#   - Build multiple subgrouping variables and run simple LM:
#       log2(abundance + eps) ~ exposure_var
#   - Summarise results per pathway x subgroup with counts & FDR
#   - Save annotated CSV
# ===========================================================

# -----------------------------
# Load libraries
# -----------------------------
library(tidyverse)
library(broom)

# -----------------------------
# User settings (edit these)
# -----------------------------
pathabundance_file <- "pathAbundance.csv"
metadata_file      <- "metadata.csv"
outdir             <- "results"

pathway_col        <- "Pathway"
sample_id_col      <- "sampleID"
exposure_var       <- "CLO_status"
exposure_positive  <- "Positive"
filter_expr        <- "Mixed_infection == 'Negative'"    # set to NULL if not filtering out mixed
infections (e.g. CLO + Ct)
subgroup_cols      <- c("Sex", "Age_group", "Trachoma_grade")

# thresholds
pseudo_eps         <- 1e-6
present_threshold  <- 1e-6
min_prev_prop      <- 0.01
min_group_n        <- 3
min_pos_present    <- 3
min_neg_present    <- 3
exclude_terms      <- c("UNMAPPED", "UNINTEGRATED")
pathway_info_sep   <- ": "

# -----------------------------
# Load data
```

```
# ----------------------------
pathabundance <- read_csv(pathabundance_file, show_col_types = FALSE)
metadata      <- read_csv(metadata_file, show_col_types = FALSE)

# Apply metadata filter if specified
if (!is.null(filter_expr) && nchar(filter_expr) > 0) {
  metadata <- metadata %>% filter(eval(parse(text = filter_expr)))
}

# ----------------------------
# Remove species-resolved rows & split pathway info
# ----------------------------
community_pathways <- pathabundance %>%
  rename(PathwayRaw = !!sym(pathway_col)) %>%
  filter(!str_detect(PathwayRaw, "\\|")) %>%
  filter(!str_detect(tolower(PathwayRaw), paste(tolower(exclude_terms), collapse = "|"))) %>%
  separate(PathwayRaw, into = c("Pathway", "More_info"), sep = pathway_info_sep, extra =
"merge", fill = "right")

# ----------------------------
# Pivot to long & join metadata
# ----------------------------
sample_cols <- setdiff(names(community_pathways), c("Pathway", "More_info"))

long_df <- community_pathways %>%
  pivot_longer(cols = all_of(sample_cols), names_to = sample_id_col, values_to = "abundance")
%>%
  left_join(metadata, by = setNames(sample_id_col, sample_id_col)) %>%
  filter(!is.na(abundance)) %>%
  mutate(log2_abundance = log2(abundance + pseudo_eps))

# ----------------------------
# Build subgroup variables
# ----------------------------
make_combo <- function(df, cols) apply(df[, cols, drop = FALSE], 1, paste, collapse = "_")

df_list <- list()
for (c1 in subgroup_cols) {
  df_list[[c1]] <- long_df %>% mutate(subgroup = .data[[c1]])
}

if (length(subgroup_cols) >= 2) {
  pairs <- combn(subgroup_cols, 2, simplify = FALSE)
  for (pair in pairs) {
    nm <- paste(pair, collapse = "+")
    df_list[[nm]] <- long_df %>% mutate(subgroup = make_combo(., pair))
```

```
  }
}
if (length(subgroup_cols) >= 3) {
  trip <- combn(subgroup_cols, 3, simplify = FALSE)
  for (tri in trip) {
    nm <- paste(tri, collapse = "+")
    df_list[[nm]] <- long_df %>% mutate(subgroup = make_combo(., tri))
  }
}

all_subgroups <- bind_rows(df_list, .id = "subgroup_definition")

# -----------------------------
# Prevalence across ALL samples
# -----------------------------
pathway_prevalence <- long_df %>%
  group_by(Pathway, More_info) %>%
  summarise(n_with_pathway = sum(abundance > present_threshold), .groups = "drop") %>%
  mutate(prevalence_OK = n_with_pathway >= (length(unique(long_df[[sample_id_col]])) *
min_prev_prop))

# -----------------------------
# Fit LMs within each subgroup
# -----------------------------
lm_results <- all_subgroups %>%
  group_by(Pathway, More_info, subgroup_definition, subgroup) %>%
  nest() %>%
  mutate(
    n_in_subgroup = map_int(data, ~ n_distinct(.x[[sample_id_col]])),
    n_subgroup_with_pathway = map_int(data, ~ sum(.x$abundance > present_threshold)),
    n_pos_with_pathway = map_int(data, ~ sum(.x$abundance > present_threshold &
.x[[exposure_var]] == exposure_positive, na.rm = TRUE)),
    n_neg_with_pathway = map_int(data, ~ sum(.x$abundance > present_threshold &
.x[[exposure_var]] != exposure_positive, na.rm = TRUE)),
    model = map(data, ~ tryCatch(lm(as.formula(sprintf("log2_abundance ~ %s", exposure_var)),
data = .x), error = function(e) NULL)),
    tidy_model = map(model, ~ if (!is.null(.x)) tidy(.x) else NULL)
  ) %>%
  unnest(tidy_model) %>%
  filter(str_detect(term, exposure_var)) %>%
  left_join(pathway_prevalence, by = c("Pathway", "More_info")) %>%
  mutate(
    direction = ifelse(estimate > 0, paste0(exposure_positive, ">other"), paste0("other>",
exposure_positive)),
    p_adj = p.adjust(p.value, method = "fdr"),
    n_subgroup_OK = n_in_subgroup >= min_group_n,
```

```
    filter_status = ifelse(
      prevalence_OK & n_subgroup_OK &
        n_pos_with_pathway >= min_pos_present &
        n_neg_with_pathway >= min_neg_present,
      "PASS", "FAIL"
    )
  ) %>%
  select(
    Pathway, More_info, subgroup_definition, subgroup,
    log2_estimate = estimate, direction, p.value, p_adj,
    n_in_subgroup, n_with_pathway, n_subgroup_with_pathway,
    n_pos_with_pathway, n_neg_with_pathway, filter_status
  )

# -----------------------------
# Save annotated results
# -----------------------------
if (!dir.exists(outdir)) dir.create(outdir, recursive = TRUE)
stamp <- format(Sys.time(), "%Y%m%d_%H%M%S")
outfile <- file.path(outdir, sprintf("significant_pathways_by_subgroup_annotated_%s.csv", stamp))
write_csv(lm_results, outfile)

cat("Saved results to:", outfile, "\n")
```
